# The E-AHPBA—ESSO—Innsbruck consensus recommendations on peri- and postoperative management following liver resection

**DOI:** 10.1093/bjs/znaf272

**Published:** 2025-12-30

**Authors:** Eva Maier, Stefan Stättner, Lucia Carrion-Alvarez, Marcello Di Martino, Pim Olthof, Florian Primavesi, Dana Sochorova, Stijn van Laarhoven, Anita Balakrishnan, Robert Breitkopf, Carlijn I Buis, Federica Cipriani, Joris Erdmann, Adam Frampton, David Fuks, Stefan Gilg, Aiste Gulla, Francesco Lancellotti, Christian Margreiter, Emmanuel Melloul, Christian Oberkofler, Stefan Petritsch, Helmut Raab, Nuh N Rahbari, Daniela Rappold, Thomas Reiberger, Andrea Ruzzenente, Ville Sallinen, Benedikt Schaefer, Andreas A Schnitzbauer, Alejandro Serrablo, Kjetil Soreide, Ernesto Sparrelid, Patrick Starlinger, Gregor A Stavrou, Pascale Tinguely, Luca Aldrighetti, Bobby V M Dasari, Matteo Donadon, Cristina Dopazo, Thomas Gruenberger, Eduard Jonas, Hassan Malik, Luca Viganó, Ajith K Siriwardena, Manuel Maglione

**Affiliations:** Department of Visceral, Transplant and Thoracic Surgery, Medical University of Innsbruck, Innsbruck, Austria; Department of Visceral and General Surgery, Hepatobiliary Centre, Kepler University Hospital GmbH, Linz, Austria; Medical Faculty, Johannes Kepler University, Linz, Austria; HPB Unit, General Surgery Department, Fuenlabrada University Hospital, Madrid, Spain; Department of Health Sciences, Università del Piemonte Orientale, Novara, Italy; Division of General Surgery, Azienda Ospedaliera Universitaria Maggiore della Carità, Novara, Italy; Department of Surgery, Erasmus MC Cancer Institute, Rotterdam, The Netherlands; Department of Visceral, Transplant and Thoracic Surgery, Medical University of Innsbruck, Innsbruck, Austria; Department of Surgery, Tomas Bata Hospital, Zlin, Czech Republic; Department of Surgery, Leiden University Medical Centre, Leiden, The Netherlands; Cambridge Hepatopancreatobiliary Unit, Cambridge University Hospitals NHS Foundation Trust, Cambridge, UK; Department of Anaesthesiology and Intensive Care Medicine, Medical University Innsbruck, Innsbruck, Austria; Department of Hepato-Pancreato-Biliary Surgery and Liver Transplantation, University Medical Centre Groningen, Groningen, The Netherlands; Hepatobiliary Surgery Division, San Raffaele Scientific Institute, Milan, Italy; Department of Surgery, Amsterdam UMC, Amsterdam, The Netherlands; HPB Surgical Unit, Royal Surrey NHS Foundation Trust, Guildford, UK; Section of Oncology, Department of Clinical and Experimental Medicine, University of Surrey, Guildford, UK; Department of Visceral Surgery, Lausanne University Hospital (CHUV), Lausanne, Switzerland; Faculty of Biology and Medicine, University of Lausanne (UNIL), Lausanne, Switzerland; Department of Clinical Science, Intervention and Technology, Division of Surgery and Oncology, Karolinska Institutet, Karolinska University Hospital, Stockholm, Sweden; Faculty of Medicine, Institute of Clinical Medicine, Vilnius University, Vilnius, Lithuania; Regional Hepato-Pancreato-Biliary Unit, Manchester Royal Infirmary, Manchester, UK; Robotic and Minimally Invasive Digestive Surgery, Arcispedale Sant’Anna, Ferrara, Italy; Department of Visceral, Transplant and Thoracic Surgery, Medical University of Innsbruck, Innsbruck, Austria; Department of General Surgery, Medical University of Graz, Graz, Austria; Department of Visceral Surgery, Lausanne University Hospital (CHUV), Lausanne, Switzerland; Faculty of Biology and Medicine, University of Lausanne (UNIL), Lausanne, Switzerland; Vivévis AG—Visceral, Tumour and Robotic Surgery, Clinic Hirslanden Zurich, Zurich, Switzerland; Department for General, Visceral and Vascular Surgery, Salzkammergut Klinikum Vöcklabruck, Vöcklabruck, Austria; Department of Anaesthesiology and Intensive Care Medicine, Medical University Innsbruck, Innsbruck, Austria; Department of Surgery, Medical Faculty Mannheim, University Hospital Mannheim, University of Heidelberg, Mannheim, Germany; Department for General, Visceral and Vascular Surgery, Salzkammergut Klinikum Vöcklabruck, Vöcklabruck, Austria; Division of Gastroenterology and Hepatology, Department of Medicine III and CD-Lab for Portal Hypertension and Liver Fibrosis, Medical University of Vienna, Vienna, Austria; General and Hepatobiliary Surgery, Department of Surgery, Dentistry, Gynaecology and Paediatrics University of Verona, GB Rossi Hospital, Verona, Italy; Department of Gastroenterological Surgery, Helsinki University Hospital and University of Helsinki, Helsinki, Finland; Department of Transplantation and Liver Surgery, Helsinki University Hospital and University of Helsinki, Helsinki, Finland; Department of Gastroenterology, Medical University Innsbruck, Innsbruck, Austria; Knappschaft Clinics University Hospital Bochum, Ruhr-University Bochum, Bochum, Germany; HPB Unit, Miguel Servet University Hospital, Zaragoza, Spain; Department of Gastrointestinal Surgery, HPB Unit, Stavanger University Hospital, Stavanger, Norway; Department of Clinical Medicine, University of Bergen, Bergen, Norway; Department of Clinical Science, Intervention and Technology, Division of Surgery and Oncology, Karolinska Institutet, Karolinska University Hospital, Stockholm, Sweden; Department of Surgery, Division of Hepatobiliary and Pancreatic Surgery, Mayo Clinic, Rochester, Minnesota, USA; Centre of Physiology and Pharmacology, Medical University of Vienna, Vienna, Austria; Department of General, Visceral and Thoracic Surgery, Surgical Oncology, Klinikum Saarbruecken, Saarbruecken, Germany; Department of Visceral and Transplantation Surgery, University Hospital Zurich, University of Zurich, Zurich, Switzerland; Hepatobiliary Surgery Division, San Raffaele Scientific Institute, Milan, Italy; Department of HBP and Liver Transplantation Surgery, University of Birmingham, Birmingham, UK; Department of Health Sciences, Università del Piemonte Orientale, Novara, Italy; Division of General Surgery, Azienda Ospedaliera Universitaria Maggiore della Carità, Novara, Italy; Department of General Surgery, Division of HPB Surgery and Transplants, Vall d’Hebron Hospital Universitari, Vall d’Hebron Institut de Recerca (VHIR), Universitat Autónoma de Barcelona Vall d’Hebron Barcelona Hospital Campus, Barcelona, Spain; Department of Surgery, Clinic Favoriten, HPB Centre, Health Network Vienna and Sigmund Freud Private University, Vienna, Austria; Surgical Gastroenterology Unit, Division of General Surgery, Department of Surgery, Faculty of Health Sciences, University of Cape Town, Cape Town, South Africa; Liver Surgery Unit, Liverpool University Hospitals NHS Foundation Trust, Liverpool, UK; Department of Biomedical Sciences, Humanitas University, Pieve Emanuele, Italy; Hepatobiliary Unit, Department of Minimally Invasive General and Oncological Surgery, Humanitas Gavazzeni University Hospital, Bergamo, Italy; Regional Hepato-Pancreato-Biliary Unit, Manchester Royal Infirmary, Manchester, UK; Department of Visceral, Transplant and Thoracic Surgery, Medical University of Innsbruck, Innsbruck, Austria

## Abstract

**Background:**

Liver surgery carries a high risk of complications due to the complex interplay of patient-related factors, disease characteristics, and liver function. Expertise is essential for healthcare professionals managing hepatobiliary patients. This European consensus provides evidence-based guidance on selected aspects of peri- and postoperative care.

**Methods:**

A modified Delphi process was used to achieve consensus, with a 70% agreement threshold. The expert panel comprised hepatobiliary surgeons, anaesthetists, hepatologists, a specialist nurse, and a physiotherapist. A systematic literature search was conducted in PubMed/MEDLINE, Embase, Web of Science, and Cochrane databases. Six topics were addressed: thromboprophylaxis; perioperative antibiotics; prehabilitation/nutrition/mobilization; bile leak including bilioenteric anastomosis leaks; post-hepatectomy haemorrhage; and post-hepatectomy liver failure (PHLF). Evidence appraisal and statement development followed Scottish Intercollegiate Guidelines Network methodology. A patient representative reviewed the guideline.

**Results:**

Searching the literature yielded 204 included publications from an initial 6514. Thirty-two statements were formulated with a median evidence level of 2. Evidence strength varied by topic with noticeably lower evidence levels in complex surgery settings and less prevalent conditions. In some topics, study heterogeneity and specific inclusion criteria resulted in conditional recommendations, despite high-level evidence. Notably, the weakest evidence was found for perioperative thromboprophylaxis and PHLF management. Strong recommendations were formulated for prehabilitation, early postoperative mobilization, and avoidance of routine drain use. Several evidence gaps warranting multicentre studies were identified.

**Conclusion:**

Optimizing peri- and postoperative care after liver resection remains challenging. Standardizing key practices and addressing evidence gaps through collaborative research are vital to improve outcomes.

## Introduction

Liver resection is a fundamental intervention for the treatment of primary and secondary hepatic malignancies, as well as selected benign liver disorders. Advances in surgical technique and perioperative care have improved outcomes. Liver resections are still characterized however by a complex interplay of patient-, malignancy-, and liver function-related factors, resulting in a high risk of complications. These include bile leaks, haemorrhage, venous thromboembolism (VTE), surgical-site infections (SSIs), and post-hepatectomy liver failure (PHLF) eventually leading to worsened short- and long-term outcomes^1–5^.

Despite growing clinical experience, evidence guiding peri- and postoperative management is frequently of moderate quality, largely derived from retrospective studies, institutional protocols, or expert opinion.

Professional societies such as the ERAS^®^ (Enhanced Recovery After Surgery) Society have published guidelines with the aim of optimizing perioperative patient management^6^. Regarding prevention and management of post-hepatectomy complications, to date, no consensus recommendations guiding patient care are however available.

Considering the persistent inconsistency in clinical practices, the overarching objective of this consensus project was to furnish clinical guidance on specific perioperative matters and the management of postoperative complications, categorized in six topics.

Perioperative thromboprophylaxis, its timing and duration, and the management of occurring portal vein thrombosis (PVT) was addressed. In the context of liver resections, there is often a delicate balance to be considered between the risk of bleeding and the occurrence of thrombotic complications^7^. This is further complicated by hypercoagulability during the perioperative period^8^.

In light of the safe, abbreviated perioperative regimens and reduced SSIs following tailored antibiotic administration^9,10^, the second topic covered perioperative antibiotics, including recommendations on timing and length of antibiotic prophylaxis for standard liver resections and those for patients considered at high risk.

Given the expansion of boundaries in liver resection, the importance of preoperative multimodal prehabilitation protocols is increasing^6,11^. Themes pertaining to preoperative screening and perioperative nutritional optimization were selected for this consensus.

Postoperative bile leakage is a prevalent complication, with incidence rates ranging from 5% to 15% and even higher following perihilar resections^12,13^. The fourth topic included issues pertaining to leakage prevention, early detection, and treatment strategies.

Then post-hepatectomy haemorrhage (PHH) was addressed. Rather than emphasizing the management of this life-threatening complication, which is undoubtedly influenced by patients’ conditions^13,14^, this consensus focused on various pre- and intraoperative strategies designed to avert this deleterious complication.

Finally, this consensus focused on diagnosis, management, and monitoring of patients experiencing PHLF following the recently published Innsbruck consensus on preoperative liver function assessments^15^.

The objective of this European consensus project was to harmonize practices across these six domains. It should guide clinicians through evidence-informed recommendations for optimal peri- and postoperative care following liver resection.

## Methods

### Selection of committee members

The steering committee (E.M., M.M., A.S., S.S.) selected 46 members for the expert panels, validation committee, and writing group based on their experience and expertise in the field of pre-, intra-, and postoperative management of liver surgery patients and on their scientific contribution on challenges surrounding liver resections and complication management. Care was taken to ensure a balanced mix of participants’ characteristics in terms of demographics (age, sex, country), clinical specialty, and experience. The consensus development involved a broad variety of specialties (hepatobiliary surgeons, anaesthetists, hepatologists, a specialist nurse, and a physiotherapist) and was well balanced in terms of age and sex, and also nationalities with input from 21 different countries. All individuals made a declaration of interest regarding potential financial and non-financial conflicts related to the consensus recommendation content.

### Selection of key topics to develop in the consensus

After an exploratory PubMed literature review by the steering committee and following several online meetings (October 2023), six key areas were identified covering different topics on peri- and postoperative management following liver resection. These included: perioperative thromboprophylaxis; perioperative antibiotics; prehabilitation/nutrition/mobilization; post-hepatectomy bile leak including bilioenteric anastomosis leaks; PHH; and PHLF. Each area was further stratified into subtopics.

The experts were assigned to individual groups according to their scientific and clinical profile. Under the direction of elected group leaders, each group could either perform a systematic literature search by itself or was provided with the results of a centralized literature search querying PubMed/MEDLINE, Embase, Web of Science, and Cochrane databases between January 2010 and February 2024 according to pre-specified Medical Subject Heading (MeSH) search terms, expanded by individual keywords related to subtopics. Publications deemed to be pivotal to the subject matter, but not supplied by the centralized search due to their publication date were also included following a careful review by the expert panel. The publications were prefiltered according to inclusion and exclusion criteria (*supplementary methods* and *Table S1*).

### Methodology for guideline development

The methodology followed a previously described process^16^. Three validated methods were integrated including SIGN (Scottish Intercollegiate Guidelines Network) methodology for the assessment of evidence and development of guideline statements^17^, the modified Delphi method for achieving expert consensus^18,19^, and the AGREE II-GRS (Global Rating Scale) instrument for assessment of methodological quality and externally validating final statements^20^.

### Inclusion and grading of evidence

Included studies were assessed and graded according to SIGN methodology by evaluating the ‘study quality’ and ‘evidence level’ according to the SIGN grading system (*Tables S2*, *S3*). The resulting ‘evidence tables’ were then reviewed by the steering committee to ensure correct application of the inclusion and exclusion criteria and gradings. The working groups then created ‘considered judgement forms’ to summarize the evidence, quality ratings, limitations, and strength of individual studies, resulting statements, strength of recommendations, and future areas of scientific interest.

### Modified Delphi process

The key questions and proposed guideline statements with attached judgement forms were sent out for a stepwise Delphi process for anonymous voting to the whole expert panel. It allowed every member to agree or disagree with the statement and to make comments and recommendations for changes. An agreement level of 70% per Delphi stage was considered appropriate to ensure a balance between consensus and voting progress. In three Delphi rounds, statements reaching 70% agreement were excluded from further discussion, while statements below this level were reviewed by the steering committee and working group leaders and revised accordingly to enter the next Delphi round. At the final virtual preconference meeting the remaining statements were again discussed, reviewed, and then adopted for the onsite conference. This conference was held as part of the Surger-I-nnsbruck International Meeting on Liver Surgery on 12–13 December 2024 in Innsbruck, Austria. Here, all key questions, statements, and preliminary paragraphs drafted by the six groups were summarized with their underlying evidence and presented to the committee and the audience. All congress participants could discuss and ultimately vote electronically on their individual support for the statements. The presentations, discussions, and voting results were reviewed by the validation committee, which provided final thoughts and comments on each statement before endorsing it. This final voting of the conference participants and the validation committee was considered by the writing and steering committee for refining the wording of individual statements and expert comment paragraphs for the published manuscript.

### Validation

The entire guideline process was quality-controlled by the validation committee (*supplementary Figures*). All members of the panel and a patient representative (Gerhard Lobenscheg, patient support group ‘L(i)eberleben’, Tyrol, Austria) reviewed the draft before submitting it for publication. The editorial staff of the publisher was involved during the process in terms of formal structure and layout. The consensus development was endorsed by the Scientific and Research Committee of the European-African Hepato-Pancreato-Biliary Association (E-AHPBA) and the Board of Directors of the European Society of Surgical Oncology (ESSO).

## Results

A total of 6514 publications was reviewed by the six working groups, resulting in a final number of 204 included manuscripts (*supplementary results*).

After two online Delphi rounds all but one statement had achieved 70% agreement level. This one was reviewed, discussed, and then finally included after reaching sufficient agreement during the onsite conference. All statements (*n* = 32) were supported by the validation committee and included in the paper after final revision. The overall methodological quality of the guideline development was considered 6.6 to 7.0 by all members of the validation committee (*supplementary Figures*).

Each clinical question is answered with a short overall statement, including the grade of recommendation and the level of evidence, followed by an expert panel comment including a selection of relevant publications related to the question. A complete list of references for each topic can be found in the *supplementary results*. An outlook for future research questions has been generated (*Table 1*).

**Table 1:** Priority areas for future research

Area	Rationale and unmet needs
Thromboprophylaxis following portal vein reconstruction and liver resection	There is currently no strong evidence on specific anticoagulation protocols after portal vein reconstruction and hepatectomy available. In trials, patients at risk for PVT should be defined and effectiveness and costs regarding duration of thromboprophylaxis and pharmacological agents (for example LMWH *versus* unfractionated heparin) should be compared.
PVT classification	The application of the 30-day threshold (according to transplant surgery) is pragmatic but arbitrary. Studies should focus on a possible pathophysiological distinction between early and late PVT. Definitions are needed that include time point, extent, and duration of PVT to optimize intervention and surveillance strategies.
Optimal duration of perioperative antibiotic therapy	There is uncertainty about sufficiency of short-course *versus* prolonged antibiotic regimens. Future studies should identify preoperative, intraoperative, and postoperative risk factors to develop predictive models and to investigate potential cost-saving effects of different treatment regimens.
Prehabilitation	Standardized metrics for nutritional risk or optimal prehabilitation components are lacking. Trials should focus on assessment tools to improve patient selection and outcomes and address economic challenges.
Bile leak risk modelling	Complex hepatectomies with biliary resections are associated with high morbidity. Studies aimed at risk modelling could guide selective drainage and imaging postoperatively.
Anastomotic stenting	There is no evidence supporting the use of transanastomotic stents even though it is still common practice in high-risk anastomoses. Predictive models based on preoperative, intraoperative, and postoperative risk factors need to be developed and potential cost-saving effects when applying stenting selectively in patients at high risk of bile leakage should be investigated.
Biomarker-based prediction of PHLF	Several markers are currently available and in clinical use. Comparative studies are needed to define the best suitable biomarker for clinical practice.

PVT, portal vein thrombosis; LMWH, low molecular weight heparin; PHLF, post-hepatectomy liver failure.

## Topic 1: perioperative thromboprophylaxis

*Q1 When should perioperative thromboprophylaxis be commenced in liver surgery?*

Pharmacological thromboprophylaxis is recommended for all patients undergoing liver surgery and can be initiated either pre- or postoperatively. Mechanical thromboprophylaxis (socks, pneumatic) should be considered routinely.

*Recommendation grade:* conditional | median level of evidence: 2+ (range: 2++ to 3)

**Expert panel comment**

The incidence of post-hepatectomy VTE has been reported to range between 2% and 6%, which is higher than the incidence observed for many other abdominal surgeries and is directly proportional to the magnitude of the hepatectomy, with extended hepatectomies exhibiting the highest risk^21^. Published hepatectomy bleeding event rates range from 1% to 8%^13^, with most series focusing their data on intraoperative bleeding and transfusion rates. An American College of Surgeons National Surgical Quality Improvement Program (ACS-NSQIP) analysis reported that the gradient between VTE event rate and bleeding complications ranged from 3.5 to 8.3 times and increased significantly with the magnitude of hepatectomy^21^.

Pharmacological thromboprophylaxis with low molecular weight heparin (LMWH) or unfractionated heparin should be initiated as a standard postoperative care measure, unless deemed contraindicated by exceptional circumstances^7^. There is no high-quality evidence to guide the optimal timing of thromboprophylaxis in liver surgery specifically. In particular, evidence is lacking on whether it should be initiated preoperatively or postoperatively^22^.

Retrospective uncontrolled studies have provided low-quality evidence suggesting that preoperatively initiated pharmacological thromboprophylaxis using LMWH could decrease VTE events^21,23^. However, regarding bleeding, the reports are conflicting. Therefore, the current clinical practice is highly variable between centres^24^. While postoperatively initiated thromboprophylaxis is associated with lower VTE rates without an increase in bleeding complications, no strong recommendation can be made in the preoperative setting. An RCT (PREPOSTEROUS trial, NCT04731558) comparing preoperative to postoperative thromboprophylaxis in liver surgery is currently recruiting^25,26^. Until high-quality evidence emerges, either approach is acceptable.

*Q2 What is the optimal duration of perioperative thromboprophylaxis in liver surgery?*

A 4-week period of administration of LMWH should be considered after major liver surgery. Prolonged (>4 weeks) prophylaxis may however be indicated in high-risk patients based on individual risk stratification.

*Recommendation grade:* conditional | median level of evidence: 2− (range: 2− to 4)

**Expert panel comment**

High-level evidence for perioperative thromboprophylaxis specifically in liver surgery, adjusted to patient’s factors, extent of surgery, and open *versus* minimally invasive approach is lacking. The term ‘prolonged’ prophylaxis is used variably in the literature, referring to 4 weeks or more than 4 weeks, depending on the study. Additionally, most of the data are derived from the in-hospital stay with limited information on post-discharge VTE and efficacy of its prevention. Since there are studies indicating an increased risk of VTE after liver surgery compared to other oncological surgery, many advocate prolonged prophylaxis of 4 weeks to all patients undergoing liver surgery for malignancy and this seems to be feasible without increased risk of bleeding complications. Others only recommend prolonged duration to high-risk patients using individual risk stratification tools^27^ (for example Padua prediction score^28^) to define high-risk patients. Antithrombin III (AT III) deficiency and PHLF must be considered as confounding factors. Liver resections of a limited extent performed by minimally invasive approach carry less risks and these patients could be considered for less than 4 weeks of LMWH, unless individual other risk factors exist related to chronic conditions.

*Q3 What anticoagulation should be used when performing hepatectomy with portal vein resection and reconstruction?*

There is no strong evidence for specific perioperative prevention strategy of PVT in patients undergoing liver surgery with portal vein resection and reconstruction. The approach should be tailored according to the patient-specific factors and clinical judgement.

*Recommendation grade:* conditional | median level of evidence: 2− (range: 1+ to 2−)

**Expert panel comment**

In 2016, Miyazaki *et al*.^29^ analysed 270 patients who underwent a portal vein resection (including end-to-end anastomosis, patch or segmental graft, and venorrhaphy) after 74 hepatectomies and 196 pancreatectomies. They introduced a systemic heparinization (200–240 units/kg/day) from the first postoperative day to 7 postoperative days. They recorded postoperative PVT in 24 patients (8.9%), 16 out of 74 patients (21.6%) after hepatectomy and 8 out of 188 patients (4.3%) after pancreatectomy. Molina *et al*.^30^ reported a policy of a prophylactic LMWH to all patients who underwent hepatectomy for perihilar cholangiocarcinoma, moving to administration every 12 h for patients with vascular resection. Considering lack of evidence on this topic, a tailored approach may be applied according to patient-specific factors. Ejaz *et al*.^31^ found that patients with previous history of VTE, prolonged operative time (OR 1.17 per additional hour (95% c.i. 1.04 to 1.32); *P* = 0.009) and increased length of stay were independent risk factors for VTE. Unfortunately, no direct mention has been made regarding PVT in patients who received a vascular resection and reconstruction during hepatectomy.

*Q4 Is there a role for defining early and late PVT after liver resection?*

Defining early and late PVT after liver resection should be considered with the aim to standardize outcomes. A widely accepted definition specific to liver resection is lacking. A 30-day cut-off, as used in liver transplantation, is a pragmatic approach that may be adopted, with limitations related to partially different pathophysiological causes of PVT after liver resection compared to liver transplantation.

*Recommendation grade:* conditional | median level of evidence: no evidence available

**Expert panel comment**

Early PVT is typically related to immediate postoperative factors, such as direct surgical manipulation or technical failure, intraoperative haemodynamic changes, or pro-thrombotic conditions. The detection of very early postoperative PVT would necessitate a routine imaging protocol, such as Doppler ultrasonography within 48 h of major liver surgery. Late PVT, occurring after 30 days, often reflects long-term processes, including liver regeneration and altered portal venous flow. Proposed post-resection PVT classification system^32^ include categories according to the site of the thrombus but do not reflect the time aspect. The decision for a 30-day cut-off is a practical solution to distinguish these phases but remains somewhat arbitrary and may require further validation. Defining PVT according to the time of onset may be useful for academic purposes. From a clinical point of view, the cut-off should also consider the management strategy for PVT, such as anticoagulation or invasive interventions, as these approaches may differ based on the timing, location, and nature of the thrombosis. Early PVT may more often require reoperation, late PVT is often asymptomatic, detected on follow-up imaging and can be mainly managed by anticoagulation.

*Q5 Are there differences in the treatment of early and late PVT after liver resection?*

There are no high-quality comparative studies to establish standardized treatment algorithms for early and late PVT after liver resection. Treatment options include anticoagulation, surgical intervention, and interventional radiology procedures. The decision on treatment strategy should be adjusted according to the time onset, thrombus extent and site, patient-specific factors, and institutional resources.

*Recommendation grade:* conditional | median level of evidence: no evidence available

**Expert panel comment**

Early PVT poses a significant risk of complications such as liver failure, portal hypertension, or bowel ischaemia and requires timely intervention. Treatment strategies depend not only on the extent (partial *versus* total), timing (early *versus* late), and duration of existence of the thrombosis, but also on the site and on the patient’s conditions at the time of diagnosis. A surgical approach can be considered for early thrombosis at the site of reconstruction, as one of the possible causes is a technical factor following the index procedure. The administration of anticoagulation aims to prevent thrombosis extension and related decrease in portal venous flow, which may result in portal hypertension and liver failure. Late PVT management focuses on addressing long-term complications, such as chronic portal hypertension, with tailored anticoagulation strategies based on follow-up imaging. While institutional resources play a crucial role in determining the feasibility of specific interventions, such as advanced interventional radiology or surgical expertise, there remains a significant gap in high-level evidence to support specific recommendations.

**Topic 1:** Perioperative thromboprophylaxis

Subtopic	Recommendation	Recommendation grade	Median level of evidence
Q1: When should perioperative thromboprophylaxis be commenced in liver surgery?	Pharmacological thromboprophylaxis is recommended for all patients undergoing liver surgery and can be initiated either pre- or postoperatively. Mechanical thromboprophylaxis (socks, pneumatic) should be considered routinely.	Conditional	2+
Q2: What is the optimal duration of perioperative thromboprophylaxis in liver surgery?	A 4-week period of administration of LMWH should be considered after major liver surgery. However, a prolonged (>4 weeks) prophylaxis may be indicated in high-risk patients based on individual risk stratification.	Conditional	2−
Q3: What anticoagulation should be used when performing hepatectomy with portal vein resection and reconstruction?	There is no strong evidence for a specific perioperative prevention strategy of PVT in patients undergoing liver surgery with portal vein resection and reconstruction. The approach should be tailored according to patient-specific factors and clinical judgement.	Conditional	2−
Q4: Is there a role for defining early and late PVT after liver resection?	Defining early and late PVT after liver resection should be considered with the aim to standardize outcomes. A widely accepted definition specific to liver resection is lacking. A 30-day cut-off, as used in liver transplantation, is a pragmatic approach that may be adopted, with limitations related to partially different pathophysiological causes of PVT after liver resection compared to liver transplantation.	Conditional	n.a.
Q5: Are there differences in the treatment of early and late PVT after liver resection?	There are no high-quality comparative studies to establish standardized treatment algorithms for early and late PVT after liver resection. Treatment options include anticoagulation, surgical intervention, and interventional radiology procedures. The decision on treatment strategy should be adjusted according to the time of onset, thrombus extent and site, patient-specific factors, and institutional resources.	Conditional	n.a.

LMWH, low molecular weight heparin; PVT, portal vein thrombosis.

## Topic 2: perioperative antibiotics

*Q1 Which antibiotics should be used as standard prophylaxis for liver surgery?*

For liver surgery prophylaxis, antibiotic choice should align with local pathogen resistance patterns. First-generation cephalosporins such as cefazolin are preferred for livers with naive bile ducts, administered within 60 min before incision. Broader-spectrum agents, like ceftriaxone or piperacillin/tazobactam, are conditionally recommended for high-risk cases. Consultation with a microbiologist should be considered in methicillin-resistant *Staphylococcus aureus* (MRSA)-risk settings. In β-lactam allergic patients, fosfomycin or aminoglycosides should be selected.

*Recommendation grade:* conditional | median level of evidence: 2++ (range: 1++ to 2+)

**Expert panel comment**

Guidelines for prophylactic antibiotics in liver surgery emphasize the need to balance infection prevention with antibiotic stewardship. Currently, there is no conclusive evidence to either support or refute the routine use of antibiotic prophylaxis for all liver resections^33,34^. Cefazolin, a first-generation cephalosporin, is however recommended for standard liver resections due to its effectiveness in reducing SSIs and its minimal impact on 30-day readmission rates. Administered 1 h before surgery, cefazolin provides reliable coverage for clean-contaminated procedures, addressing the infection risk in most patients undergoing liver resection^35,36^.

For higher-risk patients, broader-spectrum antibiotics like third-generation cephalosporins or β-lactam/β-lactamase inhibitors may be appropriate, providing enhanced coverage against Gram-negative and anaerobic pathogens. The decision to use these alternatives should consider patient risk factors and local resistance trends. Although effective, these agents are recommended conditionally, given the moderate-quality evidence supporting their use in specific cases^37–43^.

For patients at risk of MRSA infection, adding vancomycin to the prophylactic regimen is suggested, but should be guided by local resistance data and individual patient factors as well as consultation with an infectious disease specialist to prevent resistance development. Aminoglycosides are reserved due to nephrotoxicity concerns and are recommended only for cases with specific coverage needs. This framework reflects a careful, evidence-based approach prioritizing effective prophylaxis while mitigating antibiotic resistance risks^44,45^.

*Q2 Which patients should be defined as high risk for developing clinically relevant SSI following hepatectomy?*

Patients at higher risk of SSI can be characterized by bacterial colonization resistant to commonly used prophylactic antibiotics or by factors which make them generally more at risk for postoperative complications like underlying liver disease, preoperative biliary drainage, and synchronous resections of the gastrointestinal tract. In patients who undergo preoperative biliary drainage, cultures should be obtained at the time of the intervention. In case of bile duct resection and reconstruction, intraoperative biliary cultures should be obtained during the surgical procedure.

*Recommendation grade:* conditional | median level of evidence: 2+ (range: 1++ to 2+)

**Expert panel comment**

Several risk factors are associated with a higher risk of developing SSI following hepatectomy. Most of the evidence comes from multivariable analyses of retrospective observational studies, with very few RCTs addressing this issue^43^. Patients at high risk for developing clinically relevant SSIs after hepatectomy can be categorized based on the preoperative, surgical, and postoperative risk factors.

Preoperative risk factors (moderate to high level of evidence) include patient-related factors (obesity, diabetes mellitus, chronic pulmonary diseases, and chronic cardiac diseases^46,47^, preoperative anaemia, malnutrition, or hypoalbuminemia^48^, colonization with bacteria resistant to commonly used perioperative antibiotics^37,46,49^), liver-related factors (hepatolithiasis, chronic liver disease, biliary obstruction, or preoperative biliary drainage^50–53^) and technical or logistic factors (previous liver resection, planned bilioenteric anastomosis^37,54,55^, prior hospitalization^49,52^).

Surgical risk factors (moderate level of evidence) comprehend high volumes of intravenous fluids, blood transfusion, prolonged operation time, and concomitant bowel surgery^37,48,51,52,55^. Finally, persistent postoperative elevation of inflammatory markers such as C-reactive protein (CRP) or bile leakage^56,57^ are examples of postoperative risk factors (moderate level of evidence).

Despite these risk factors, clear criteria for identifying such high-risk patients are not yet well defined.

*Q3 Which patients are likely to benefit from extended antibiotic prophylaxis?*

Although antibiotic prophylaxis is recommended, extended postoperative antibiotic prophylaxis for more than 24 h has not demonstrated any additional benefit in preventing postoperative infections after standard hepatectomies. Patients at high risk for developing clinically relevant SSI may benefit from extended antimicrobic duration (3–5 days postoperatively).

*Recommendation grade:* conditional | median level of evidence: 2+ (range: 1+ to 2+)

**Expert panel comment**

Data derived from RCTs are mainly available for standard hepatectomies. There is lack of evidence supporting routine extended (3–5 days postoperatively) antibiotic prophylaxis after standard liver resection, aligning with principles of antibiotic stewardship^34,58–61^. Patients at high risk for developing clinically relevant SSI may benefit from extended antimicrobic duration. The emphasis on tailoring prolonged prophylaxis to high-risk patients, particularly those with preoperative biliary drainage, or complex procedures, is consistent with findings from recent studies^40,43,62,63^. However, the recommendation would benefit from a clearer stratification of risk factors and more specific guidance on how intraoperative cultures should influence postoperative antimicrobial strategies. In addition, defining indications (for example bile contamination, duration of surgery, or intraoperative findings) for initiating extended prophylaxis would enhance clinical applicability. Future prospective studies are needed to refine high-risk criteria and validate targeted protocols^64^.

*Q4 What is the role of antibiotic treatment in cases of postoperative fever of unknown origin and no suspicion of infection or sepsis following liver resection?*

Antibiotic therapy is not routinely recommended for postoperative fever of unknown origin if diagnostic work-up excluded any signs of infection or sepsis. Patients at high risk—such as those with preoperative biliary drainages, bilioenteric anastomosis, immunosuppression, or co-morbidities like diabetes mellitus—may benefit from targeted antibiotic therapy, if early signs of infection are present.

*Recommendation grade:* strong | median level of evidence: 1+ (range: 1++ to 2+)

**Expert panel comment**

Postoperative fever after hepatectomy can arise from both infectious and non-infectious causes. Routine use of antibiotics for fever of unknown origin is generally discouraged due to the risk of promoting multidrug-resistant bacteria and complicating future antibiotic selection for septic events^43,45,58^. If indicated, empirical antibiotic treatment should be guided by the severity of infection, patient-specific risk factors, and local antibiotic sensitivity patterns, covering common pathogens like *Enterococcus* species and Gram-negative bacteria until specific pathogens are identified^36,43,60^. Assessing preoperative bacterial colonization in patients with biliary abnormalities is crucial and is supported by high-level evidence, aiding in implementing targeted antibiotic strategies when necessary^33,36,41^. This tailored approach aims to improve perioperative outcomes in selected high-risk patients while minimizing the development of antibiotic resistance.

**Topic 2:** Perioperative antibiotics

Subtopic	Recommendation	Recommendation grade	Median level of evidence
Q1: Which antibiotics should be used as standard prophylaxis for liver surgery?	For liver surgery prophylaxis, antibiotic choice should align with local pathogen resistance patterns. First-generation cephalosporins such as cefazolin are preferred for livers with naive bile ducts, administered within 60 min before incision. Broader-spectrum agents, like ceftriaxone or piperacillin/tazobactam, are conditionally recommended for high-risk cases. Consultation with a microbiologist should be considered in MRSA-risk settings. In β-lactam allergic patients, fosfomycin or aminoglycosides should be selected.	Conditional	2++
Q2: Which patients should be defined as high risk for developing clinically relevant SSI following hepatectomy?	Patients at higher risk of SSI can be identified by bacterial colonization resistant to commonly used prophylactic antibiotics, as well as by factors which make them generally more at risk for postoperative complications like underlying liver disease, preoperative biliary drainage, and synchronous resections of the gastrointestinal tract. In patients with preoperative biliary drainage, cultures should be obtained at the time of the intervention. In case of bile duct resection and reconstruction, intraoperative biliary cultures should be obtained during surgery.	Conditional	2+
Q3: Which patients are likely to benefit from extended antibiotic prophylaxis?	Although antibiotic prophylaxis is recommended, extended postoperative antibiotic prophylaxis for more than 24 h has not demonstrated any additional benefit in preventing postoperative infections after standard hepatectomy. Patients at high risk for developing clinically relevant SSI may benefit from extended antibiotic duration (3–5 days postoperatively).	Conditional	2+
Q4: What is the role of antibiotic treatment in cases of postoperative fever of unknown origin and no suspicion of infection or sepsis following liver resection?	Antibiotic therapy is not routinely recommended for postoperative fever of unknown origin, if diagnostic workup has excluded any signs of infection or sepsis. High-risk patients—such as those with preoperative biliary drainages, bilioenteric anastomosis, immunosuppression, or co-morbidities like diabetes mellitus—may benefit from targeted antibiotic therapy, if early signs of infection are present.	Strong	1+

MRSA, methicillin-resistant *Staphylococcus aureus*; SSI, surgical-site infection.

## Topic 3: prehabilitation/nutrition/mobilization

*Q1 What is the value of a prehabilitation programme in patients undergoing hepatectomy?*

Multimodal prehabilitation programmes (exercise training, nutritional optimization, psychological interventions) should be considered for patients prior to hepatectomy for both benign and malignant liver disease following assessment to reduce postoperative morbidity and length of hospital stay.

*Recommendation grade:* strong | median level of evidence: 1+ (1++ to 2++)

**Expert panel comment**

Prehabilitation programmes (including physical exercise training, diet optimization, psychological interventions, smoking cessation, alcohol cessation) may have positive impact on outcomes after hepatectomy, especially for malignant liver disease (30-day morbidity reduced: trend^65,66^, no effect^67,68^; length of hospital stay shortened: significant^69^, trend^65,66^, no effect^67,68,70^; length of intensive care stay shortened^67^; quality of life (QoL) improved^71^). No effect of prehabilitation on postoperative mortality was shown in two meta-analyses^68,70^. The content of prehabilitation programmes evaluated for liver or hepatopancreatobiliary surgery is heterogeneous, with one RCT evaluating exercise training after cardiopulmonary exercise testing (CPET) as a unimodal intervention^72^ and four meta-analyses and three RCTs evaluating multimodal programmes including in-hospital or home-based physical exercise training, dietary interventions, or psychological support^65–69,73,74^.

The literature is inconsistent on the duration of a prehabilitation programme, with recently published guidelines suggesting 3–6 weeks before major oncological surgery^75^. For exercise training prior to liver surgery, a minimum of 4 weeks preoperatively has been evaluated in five meta-analyses and RCTs^65,70,71,73,74^. Alcohol and smoking cessation as part of prehabilitation is recommended for surgery in general^75^, with at least 4 weeks of cessation prior to hepatectomy^6^. Due to the heterogeneity of the outcomes assessed in the included studies, no clear recommendation can be made, indicating the need for further standardized research to assess prehabilitation in liver surgery.

*Q2 What is the influence of preoperative body composition on outcomes in patients undergoing hepatectomy?*

Preoperative body composition influences outcomes in patients undergoing hepatectomy for various conditions of liver malignancies. Therefore, preoperative screening for low skeletal muscle mass (LSMM) should be done in every patient considered for hepatectomy for liver malignancies.

*Recommendation grade:* strong | median level of evidence: 1+ (1+ to 2+)

**Expert panel comment**

At least seven systematic reviews have pointed out ‘sarcopenia’ or LSMM as major risk factors for overall survival (OS) and disease-free survival (DFS) in patients undergoing hepatectomy for hepatocellular carcinoma (HCC)^76–82^ and for postoperative complication rates in resection for colorectal-liver metastasis (CRLM)^79,81^. Only one high-quality meta-analysis investigated the impact of obesity on OS and DFS in patients undergoing resection for CRLM; no significant impact was found^79^. Preoperative screening for LSMM should be performed in every patient undergoing hepatectomy for liver malignancies, as it shows significant impact on postoperative outcomes. Therefore, CT scans seem to be a good option, as they are routinely used in oncological patients for staging examination and broadly accessible. As sarcopenia is defined as loss of muscle mass, reduced function, and impaired muscle strength, the term ‘sarcopenia’ should only be used if diagnosed according to the guidelines^83^. If diagnosed preoperatively, addressing LSMM and sarcopenia with nutritional support (for example protein supplementation) and physical activity should be considered^84^. There is only limited high-quality evidence for explaining the influence of obesity on outcome in patients undergoing hepatic resection, hence further research is needed.

*Q3 What is the role of fitness screening and assessment in patients undergoing hepatectomy?*

As aerobic fitness is an important modifiable risk factor and can be improved by a prehabilitation programme, screening for this risk factor can be incorporated as a routine part of the preoperative assessment for patients undergoing liver surgery, particularly for those with borderline or reduced fitness, as it provides critical information for optimizing perioperative care and improving postoperative outcomes.

*Recommendation grade:* conditional | median level of evidence: 1- (1− to 2+)

**Expert panel comment**

Fitness assessment allows for risk stratification and the identification of potential modifiable risk factors^85^. Aerobic fitness has a strong relationship with postoperative outcomes^86–89^.

Ideally it is measured by CPET, alternatively by Steep Ramp Test (STRT), Six-Minute Walk Test (6MWT), or Thirty-Second Sit-to-Stand Test (30ST)^90–95^.

Lower CPET-derived thresholds, specifically Anaerobic Threshold values below 9.0–11.5 ml/kg/min and Volume of Oxygen peak below 15 ml/kg/min, have been associated with increased risks of postoperative complications, longer hospital stays, and poorer OS^72,89,95,96^. These markers serve as reliable predictors for identifying high-risk patients and are used to guide clinical decisions regarding the need for prehabilitation interventions and perioperative management.

CPET offers superior predictive accuracy compared to simpler tests such as the Incremental Shuttle Walk Test (ISWT) or the STRT, which have shown limited precision in predicting postoperative outcome^95,96^. Despite their practicality, these alternative tests are not as sensitive or specific as CPET in evaluating fitness for liver surgery.

*Q4 Does perioperative immunonutrition offer a benefit in patients undergoing hepatectomy?*

Immunonutrition can be administered as perioperative nutritional supplementation in patients undergoing hepatectomy as it has a positive effect on postoperative liver function and might foster the regenerative potential especially in vulnerable populations.

*Recommendation grade:* conditional | median level of evidence: 1− (1++ to 2+)

**Expert panel comment**

A review of nine RCTs with 966 patients, including administration of ω-3-fatty acids (FAs), arginine/glutamine, and RNA applications, found improvement in complication rates, overall postoperative infectious complications, and wound infections and a beneficial effect on the length of hospital stay^97^.

No effects of immunonutrition could be found on recurrence in patients with HCC^98^. In an RCT of 320 patients undergoing resection for HCC, the intervention group, however, profited significantly from postoperative parenteral administration of ω-3-FAs with regard to complications and infections^99^.

A meta-analysis confirmed the findings in 11 RCTs (>1000 patients) with an improved clinical course reflected by lower complications^100^. The timing of the intervention was heterogeneous (pre/post), as well as the duration, which lasted from 30 days preoperatively until 3 weeks postoperatively. Regimens were also heterogeneous and consisted of ω-3-FAs alone ± ribonucleic acids ± glutamine/arginine supplementation either enterally or fully parenterally.

*Q5 What is the role of prokinetic policies to improve time to first bowel movement/full standard diet?*

Routine use of postoperative prokinetics or laxatives is recommended to stimulate bowel movement after hepatectomy, while prokinetics may shorten the time to first flatus or stool. In patients who have had a simultaneous liver and colonic resection, use of prokinetic treatment should be used with caution and based upon established colorectal protocols.

*Recommendation grade:* conditional | median level of evidence: 1+ (1++ to 2+)

**Expert panel comment**

At least four RCTs and one case–control prospective study have shown that the use of prokinetics such as postoperative laxatives, gum chewing, or herbal medicine after hepatectomy reduce the time to first flatus or stool^101–105^. Prokinetics did however not reduce secondary outcomes, such as delayed gastric emptying, length of stay, or time to functional recovery.

While some members of the expert panel suggested that time to first flatus or stool is not a clinically relevant endpoint in liver surgery, others argued that this might have a positive impact on patients’ well-being and QoL.

*Q6 When and how should artificial nutrition be added to oral nutrition in the postoperative period?*

Early oral nutrition should be preferred after hepatectomy. In patients requiring artificial nutrition support (malnourished patients, patients with complications causing several days of fasting), enteral nutrition through a nasofeeding tube should be preferred over total parenteral nutrition (TPN).

Nutritional specialist assessment is recommended for patients with delayed oral intake.

*Recommendation grade:* conditional | median level of evidence: 1− (1++ to 2++)

**Expert panel comment**

Early oral diet has been shown to be safe and to decrease the time to first bowel movement in abdominal and liver surgery^106^. The European Society for Clinical Nutrition and Metabolism (ESPEN) guidelines on clinical nutrition in surgery recommend oral nutrition postoperatively^107^.

A recent RCT of patients undergoing major abdominal surgery reported beneficial effects on nosocomial infections from early parenteral nutritional support in addition to oral intake, compared with delayed parenteral nutrition^108^. Two meta-analyses suggest that, for patients at risk of impaired oral intake over a prolonged period, enteral nutrition through a nasofeeding tube should be preferred over TPN whenever possible^109,110^.

*Q7 How should (early) postoperative mobilization after hepatectomy be defined?*

The ideal prescription on postoperative mobilization after hepatic surgery is not clear. Early mobilization after liver surgery should start immediately on postoperative day 1 with assisted active mobilization and sitting out of bed. Walking (with no precise target) should start at least on second postoperative day adapted to the patient’s functional capacity and carefully supervised especially in elderly or frail patients.

*Recommendation grade:* strong | median level of evidence: 1+ (1++ to 2+)

**Expert panel comment**

Either resistance or endurance training should be part of the mobilization for this patient population.

No literature was found on the realistic endpoints in early mobilization of elderly or frail patients. In two RCTs and two prospective cohort studies, postoperative mobilization has shown an improvement of functional capacity^111–114^. Only one study showed a reduction of the length of stay and consequently a cost-saving effect^111^. No meta-analysis on this topic was found. Two prospective cohort studies recommend activity tracking, which might detect postsurgical complications in vulnerable patients and enhance the recovery^115,116^.

Even though no study that provides a precise answer on this question was found, the authors agree that patients with a preoperative reduced level of physical activity require additional attention in the postoperative period. Especially for elderly or frail patients undergoing major surgery monitoring postoperative activity might be useful in identifying postsurgical complications. One RCT with 108 patients showed that early mobilization reduces the risk for inability to walk without human assistance at postoperative day 5 or hospital discharge^112^. No differences were however found regarding clinical outcomes or complications.

**Topic 3:** Prehabilitation/nutrition/mobilization

Subtopic	Recommendation	Recommendation grade	Median level of evidence
Q1: What is the value of a prehabilitation programme in patients undergoing hepatectomy?	Multimodal prehabilitation programmes (exercise training, nutritional optimization, psychological interventions) should be considered for patients prior to hepatectomy for both benign and malignant liver disease following assessment to reduce postoperative morbidity and length of hospital stay.	Strong	1+
Q2: What is the influence of preoperative body composition on outcomes in patients undergoing hepatectomy?	Preoperative body composition influences outcomes in patients undergoing hepatectomy for various conditions of liver malignancies. Therefore, preoperative screening for LSMM should be done in every patient considered for hepatectomy.	Strong	1+
Q3: What is the role of fitness screening and assessment in patients undergoing hepatectomy?	As aerobic fitness is an important modifiable risk factor and can be improved by a prehabilitation programme, screening for this risk factor can be incorporated as a routine part of the preoperative assessment for patients undergoing liver surgery, particularly for those with borderline or reduced fitness, as it provides critical information for optimizing perioperative care and improving postoperative outcomes.	Conditional	1-
Q4: Does perioperative immunonutrition offer a benefit in patients undergoing hepatectomy?	Immunonutrition can be administered as perioperative nutritional supplementation in patients undergoing hepatectomy as it has a positive effect on postoperative liver function and might foster the regenerative potential especially in vulnerable patients.	Conditional	1−
Q5: What is the role of prokinetic policies to improve time to first bowel movement/full standard diet?	Routine use of postoperative prokinetics or laxatives is recommended to stimulate bowel movement after hepatectomy, while prokinetics may shorten the time to first flatus or stool. In patients who have had a simultaneous liver and colonic resection, use of prokinetic treatment should be used with caution and embedded in colorectal protocols.	Conditional	1+
Q6: When and how should artificial nutrition be added to oral nutrition in the postoperative period?	Early oral nutrition should be preferred after hepatectomy. In patients requiring artificial nutrition support (malnourished patients, patients with complications causing several days of fasting), enteral nutrition through a nasofeeding tube should be preferred over TPN. Nutritional specialist assessment is recommended for patients with delayed oral intake.	Conditional	1−
Q7: How should (early) postoperative mobilization after hepatectomy be defined?	The ideal prescription on postoperative mobilization after hepatic surgery is not clear. Early mobilization after liver surgery should start immediately on postoperative day 1 with assisted active mobilization and sitting out of bed. Walking (with no precise target) should start at least on second postoperative day adapted to the patient’s functional capacity and carefully supervised especially in elderly or frail patients.	Strong	1+

LSMM, low skeletal muscle mass; TPN, total parenteral nutrition.

## Topic 4: post-hepatectomy bile leak including bilioenteric anastomosis leaks

*Q1 Is there a role for intraoperative bile leak test (that is white test, methylene blue, indocyanine green (ICG) …) after hepatectomy without bilioenteric anastomosis?*

Intraoperative bile leak tests can be considered after liver resection, especially in major resections or complex procedures, recording use in the operation report. No specific test has shown clear superiority regarding identification of leaks, although the gauze (surface) test and the ‘white test’ might be preferred. Therefore, the choice of test should depend on centre expertise and availability.

*Recommendation grade:* conditional (major/complex resections: strong) | median level of evidence: 1− (range: 1++ to 4)

**Expert panel comment**

Intraoperative bile leak testing plays an important role in identifying bile leaks at the resection plane during hepatectomy, especially after complex resections^117,118^. Bile leaks, if undetected, can lead to potentially serious complications such as bile peritonitis and intra-abdominal abscesses. There are several tests available, including the injection of different substances into the bile ducts, such as air, saline, methylene blue, lipid-based solutions (‘white test’), or ICG, standard cholangiography, and a simple stain test using gauze.

The ‘white test’ is a cost-effective technique that has been shown to reduce postoperative bile leak rates in several studies^118,119^, including two RCTs, by enabling intraoperative detection and immediate intervention^120,121^. It is important to be aware this test may not detect disconnected segments. There is an ongoing RCT comparing the white test to the gauze test, with results expected in 2025^122^.

ICG fluorescence imaging offers near-infrared visualization of bile ducts. While some retrospective studies and smaller trials suggest potential advantages of ICG in complex cases due to improved anatomical delineation^118,123^, available RCT data indicate that ICG has not shown clear superiority over simpler and more accessible techniques regarding bile leak detection rates and clinical outcomes. Despite one ongoing prospective trial^124^, further large-scale RCTs are necessary to conclusively determine the role of ICG for bile leak prevention.

*Q2 Is there a role for drainage after hepatectomy without bilioenteric reconstruction?*

Routine abdominal drainage after an uncomplicated hepatectomy should be avoided.

*Recommendation grade:* strong | median level of evidence: 1+ (range: 1++ to 2+)

**Expert panel comment**

The role of routine abdominal drainage after hepatectomy without bilioenteric reconstruction has been extensively debated, with current evidence strongly suggesting that it should be avoided in uncomplicated cases. A meta-analysis^125^, which included seven RCTs involving 1064 patients, demonstrated that postoperative drainage did not only fail to reduce the incidence of intra-abdominal collections requiring intervention but also increased the risk of overall and wound-related complications. This finding is supported by earlier publications, such as a Cochrane review^126^, which concluded that routine drainage did not provide significant benefits in uncomplicated liver resections. Similarly, there was no advantage to routine drainage after elective hepatectomy using the crush clamp method in an RCT^127^. Furthermore, Liu *et al*.^128^ highlighted that abdominal drainage is particularly contraindicated in patients with chronic liver diseases, as it may exacerbate complications without improving outcomes. Despite these findings, some specific high-risk scenarios might still warrant drain placement. Dezfouli *et al*.^129^ and Brooke-Smith *et al*.^130^ have suggested that drainage could be considered in patients with resections involving difficult anatomical locations, such as the right superior segments, or when intraoperative bile leaks are detected and concerns have risen about adequate control. Additionally, in settings where interventional radiology is not readily available, drainage may serve as a precautionary measure.

The consensus, supported by systematic reviews^131,132^, is however that routine drainage after uncomplicated hepatectomy is unnecessary and may even be harmful.

*Q3 What is the value of biliary transanastomotic stents in complex bilioenteric anastomoses in hepatectomy?*

Transanastomotic stents can be considered in complex bilioenteric anastomoses (that is extended resections, small bile ducts, friable tissue) but are not recommended for routine use. If used, they should be removed within 3 months to minimize complications. The choice of transanastomotic stent depends on centre availability and expertise.

*Recommendation grade:* conditional | median level of evidence: 2− (range: 2++ to 4)

**Expert panel comment**

Bilioenteric anastomotic leakage remains a challenging complication. It is associated with prolonged hospitalization and mortality. The decision to use transanastomotic stents in small ducts or high-risk anastomoses is still common practice aiming to reduce risk of complications. It remains however a topic of debate and stenting should be based on specific risks associated with the patient and the surgical procedure. Recent studies have identified several factors associated with bilioenteric anastomotic leakage. Risk factors can be related to the surgical procedure (complex surgeries/compromised tissue) and to patient factors (co-morbidities/previous surgery or radiation treatment)^133,134^.

Removal of these stents within 3 months is recommended to minimize side effects such as cholangitis and strictures^135^. Whether their routine use is indicated remains unclear, even if most (low evidence) studies have shown no benefit. Currently, there is no evidence supporting the use of self-resorbable transanastomotic stents.

In a large, multicentre study, preoperative biliary drainage was independently associated with a lower risk of anastomotic bile leakage, while postoperative external biliary drainage showed an association with higher leakage rates in univariable analysis. It was not an independent risk factor in multivariable analysis however^133^. Separately, the use of postoperative external biliary drains has also been identified as an independent risk factor for clinically relevant PHLF in patients undergoing major liver resection for perihilar cholangiocarcinoma^136^.

The choice of anastomotic technique has not been shown to be a relevant factor for the incidence of bile leakage in Braunwarth *et al*.^133^. Other studies underscore the value of specific surgical techniques and modifications in enhancing biliary reconstruction success, such as basin-shaped hepaticojejunostomy and a modified hepatojejunostomy technique using internal and external stents^133,137^.

*Q4 Is there a role for routine imaging (CT, magnetic resonance (MR) cholangiography) in patients at high risk for bile leaks?*

Routine use of advanced imaging modalities such as CT, MR, or single-photon emission CT (SPECT) is not recommended for asymptomatic patients at high risk due to their cost and limited availability in most healthcare settings. For patients with suspected or detected bile leaks, the threshold for performing radiological imaging should be low. The choice of modality should be guided by the resources available at the treating centre.

*Recommendation grade:* conditional | median level of evidence: 3 (range: 3)

**Expert panel comment**

Patients with suspected bile leakage originating from the resection margin or a central bile duct, whether presenting with high or low output, benefit from prompt radiological detection using, depending on local availability, ultrasound, CT scan, or more specialized imaging like bile-specific gadolinium-enhanced MR cholangiography (Gd-MR)^138–140^ or mebrofenin SPECT-CT^141^. MR cholangiography, particularly contrast-enhanced MR, is especially reliable for identifying bile leaks following hepatobiliary surgery and may reduce the need for other, potentially risky invasive diagnostic procedures^140^.

Post-hepatectomy bile leak has been defined and graded by the International Study Group of Liver Surgery (ISGLS)^130^, allowing objective comparison of the degree and severity of bile leaks. The following risk factors for postoperative bile leakage are widely accepted: preoperative risk factors—anaemia, liver cirrhosis, mechanical jaundice, chemotherapy with anti-vascular endothelial growth factor (VEGF) antibodies (bevacizumab), positive bacterial culture (bile swab), and reoperations (for reasons other than bile leakage); and intraoperative and surgical risk factors—major or complex hepatic resections including mesohepatectomies, vascular resections or reconstructions, re-resections, and central bile duct resections. Additional risk factors include operation time, blood loss, and Pringle manoeuvre (PM)^138–141^.

*Q5 What is the role of endoscopy and interventional radiology in patients with clinically relevant bile leakage after hepatectomy?*

In case of a clinically relevant bile leakage (>100 ml bile/24 h) when a drain is in place, an early endoscopic retrograde cholangiopancreatography (ERCP) and stent placement seems sensible as a strategy to resolve bile leakage quickly. When the patient has a bilioma with no drain in place, the first step should be percutaneous drainage; ERCP/stent should follow on the same conditions as above.

*Recommendation grade:* conditional | median level of evidence: 2− (1+ to 4)

**Expert panel comment**

Management of bile leakage relies strongly on locally available resources and experience. Recommendations are made in the context of healthcare systems where all available interventions are in place. This might not always be the case in clinical practice. Therefore, recruitment of external expertise might be needed occasionally. RCTs comparing early *versus* late ERCP/stent could so far not conclusively answer which approach is superior^142–148^.

**Topic 4:** Post-hepatectomy bile leak including bilioenteric anastomosis leaks

Subtopic	Recommendation	Recommendation grade	Median level of evidence
Q1: Is there a role for intraoperative bile leak test (that is white test, methylene blue, ICG *…*) after hepatectomy without bilioenteric anastomosis?	Intraoperative bile leak tests can be considered after liver resection (conditional), especially in major resections or complex procedures (strong), recording its use in the operation report. No specific test has shown clear superiority regarding identification of leaks, although the gauze (surface) test and the ‘white test’ might be preferred. Therefore, the choice of test should depend on centre expertise and availability.	Conditional (major/complex resections: strong)	1−
Q2: Is there a role for drainage after hepatectomy without bilioenteric reconstruction?	Routine abdominal drainage after an uncomplicated hepatectomy should be avoided.	Strong	1+
Q3: What is the value of biliary transanastomotic stents in complex bilioenteric anastomoses in hepatectomy?	Transanastomotic stents can be considered in complex bilioenteric anastomoses (that is extended resections, small bile ducts, friable tissue) but are not recommended for routine use. If used, they should be removed within 3 months to minimize complications. The choice of transanastomotic stent depends on centre availability and expertise.	Conditional	2−
Q4: Is there a role for routine imaging (CT, MR cholangiography) in patients at high risk for bile leaks?	Routine use of advanced imaging modalities such as CT, MR, or SPECT is not recommended for asymptomatic high-risk patients due to their cost and limited availability in most healthcare settings. For patients with suspected or detected bile leaks, the threshold for performing radiological imaging should be low. The choice of modality should be guided by the resources available at the treating centre.	Conditional	3
Q5: What is the role of endoscopy and interventional radiology in patients with clinically relevant bile leakage after hepatectomy?	In case of a clinically relevant bile leakage (>100 ml bile/24 h) when a drain is in place, an early ERCP and stent placement seems sensible as a strategy to resolve bile leakage quickly. When the patient has a bilioma with no drain in place, the first step should be percutaneous drainage; ERCP/stent should follow on the same conditions as above.	Conditional	2−

ICG, indocyanine green; MR, magnetic resonance; SPECT, single-photon emission CT; ERCP, endoscopic retrograde cholangiopancreatography.

## Topic 5: PHH

*Q1 What is the role of preoperative strategies to reduce blood loss associated with liver resection (fat-reducing diet, nutritional changes, hypovolaemic phlebotomy)?*

In patients who are overweight or obese, the implementation of a low-calorie, low-fat diet should be considered, provided that the surgical timeline permits adequate intervention. Preoperative management of metabolic dysfunction-associated steatotic liver disease (MASLD) should align with the recommendations of the European Association for the Study of the Liver (EASL)–European Association for the Study of Diabetes (EASD)–European Association for the Study of Obesity (EASO) Clinical Practice Guidelines. In selected patients at high risk of significant intraoperative bleeding, preoperative hypovolaemic phlebotomy may be considered as a strategy to reduce transfusion requirements.

*Recommendation grade:* strong | median level of evidence: 2− (range: 1++ to 3)

**Expert panel comment**

There is growing evidence that targeted preoperative strategies can reduce blood loss in patients undergoing liver resection, particularly those with MASLD.^149^ Patients with MASLD have been shown to have a 1.8-fold higher risk of postoperative bleeding, as highlighted in a recent meta-analysis^150^ underscoring the need for tailored perioperative approaches.

Nutritional interventions, especially short-term dietary restrictions, have demonstrated promising results. A small RCT found that a 1-week, 800-kcal/day diet with 20 g fat and 70 g protein significantly reduced intraoperative blood loss^151^. These findings are supported by retrospective data showing similar benefits with calorie- and fat-restricted diets^152^. Such regimens likely reduce hepatic steatosis and liver volume, improving resectability and surgical safety.

Additionally, hypovolaemic phlebotomy has re-emerged as a strategy to control intraoperative bleeding. Data of meta-analyses and the recent PRICE-2 trial both demonstrated that preoperative venesection reduces transfusion requirements without increasing complication rates^153,154^. These findings support its selective use in high-risk patients.

*Q2 What is the role of anaesthetic management to reduce blood loss in patients undergoing hepatectomy?*

Intraoperative low central venous pressure (CVP) should be maintained until completion of parenchymal transection to reduce blood loss and transfusion requirements. No specific anaesthetic agent is recommended. Tranexamic acid (TXA) is not advised in liver resection for cancer as it does not decrease blood loss during liver resection. The role of acute normovolaemic haemodilution (ANH) remains unclear.

*Recommendation grade:* moderate to strong | median level of evidence: 2++ (range: 1+ to 2−)

**Expert panel comment**

Anaesthetic expertise in liver surgery is critical in reducing intraoperative blood loss during liver resection. Data from meta-analyses of RCTs support the use of low CVP until completion of parenchymal transection. This strategy significantly reduces blood loss, shortens operating time, lowers transfusion requirements, and decreases postoperative complications, and is considered both safe and effective in adult patients undergoing hepatectomy^155^.

While certain anaesthetic agents may influence hepatic blood flow and mitigate ischaemia-reperfusion injury, particularly volatile inhalational agents metabolized by the liver, current data do not support a definitive recommendation for one agent over another in terms of bleeding outcomes. More targeted studies are needed to establish clear guidance^156^.

The use of TXA during liver resection for cancer is not supported by recent evidence. The HeLiX trial (*n* = 2145) found no reduction in intraoperative or early postoperative bleeding, transfusion needs, or VTE. Notably, TXA was associated with a higher rate of complications^157^.

The role of ANH remains uncertain. While ANH may reduce the proportion of patients requiring transfusion, it does not appear to lower total transfused volume or improve postoperative outcomes, including morbidity and mortality^155,157^.

*Q3 What is the role for intraoperative liver inflow occlusion in reducing intraoperative blood loss?*

Inflow occlusion techniques such as the PM and hemihepatic occlusion (HHO) can reduce intraoperative blood loss and should be considered when major bleeding is anticipated. While intermittent Pringle occlusion is safe for up to 120 min, prolonged clamping may increase the risk of ischaemia-reperfusion injury, particularly in patients with underlying liver dysfunction.

*Recommendation grade:* moderate | median level of evidence: 2+ (range: 1++ to 2−)

**Expert panel comment**

Intraoperative liver inflow occlusion, most commonly via the PM, is a widely adopted technique to minimize blood loss during hepatic transection. Comparative studies have evaluated PM against HHO, but findings remain mixed.

A 2011 meta-analysis of four RCTs involving 338 patients found no significant differences in intraoperative outcomes between PM and HHO, aside from higher aspartate aminotransferase (AST) levels on postoperative day 1 in the PM group, indicating transient hepatic injury^158^. A meta-analysis including eight RCTs and 688 patients published in 2019 reported significantly lower perioperative morbidity in the HHO group; no significant differences were however observed in liver-specific complications such as bile leak, PHLF, postoperative haemorrhage, and transfusion rates, but transfused volume per patient was higher in the PM group^159^.

Some evidence from a propensity-matched analysis indicated that patients with normal liver function may experience worse outcomes with PM compared to no occlusion, potentially due to ischaemia-reperfusion injury^160^.

While inflow occlusion is effective in limiting blood loss, it is not without risk. Prolonged continuous clamping can lead to ischaemia-reperfusion injury, particularly in patients with liver dysfunction. For this reason, intermittent PM—typically in 20-min cycles with 5-min reperfusion intervals—has been shown to be safe for total occlusion times up to 120 min, particularly in patients with preserved liver function^159,161^.

*Q4 Is there an influence of parenchymal transection technique on blood loss?*

Meta-analyses of RCTs suggest that bipolar energy devices may reduce intraoperative blood loss during parenchymal transection. However, due to the wide range of available techniques, the limited number of direct comparisons, and methodological variability across studies, no specific recommendation can currently be made regarding the optimal transection technique.

*Recommendation grade:* moderate | median level of evidence: 1+ (range: 1++ to 2−)

**Expert panel comment**

Parenchymal transection represents a critical phase of partial hepatectomy, with the potential to significantly influence intraoperative blood loss and postoperative complications. Multiple techniques and devices have been developed to facilitate safe and efficient transection. These range from the traditional clamp-crushing technique, which involves blunt dissection of the parenchyma with a Kelly clamp followed by clipping or ligation of vascular and biliary structures, to energy-based devices such as bipolar cautery, ultrasonic dissectors, and vascular staplers.

Over the past two decades, numerous RCTs have compared these techniques with respect to key perioperative outcomes, particularly intraoperative blood loss and postoperative complication rates—most notably bile leakage. Individual trials often yield conflicting results however due to differences in methodology and patient populations.

A 2020 systematic review and network meta-analysis of 22 RCTs sought to synthesize this evidence and found that bipolar energy devices were associated with the greatest reduction in intraoperative blood loss^162^. Nevertheless, Kamarajah *et al.*^162^ noted that cross-trial comparisons were limited by heterogeneity in surgical practices such as CVP management and use of vascular inflow occlusion. In conclusion, while certain transection techniques may offer modest advantages, particularly in reducing blood loss, the overall evidence remains inconclusive due to variability in study design and surgical context.

*Q5 What is the value of haemostatic agents to reduce blood loss in patients undergoing hepatectomy?*

No specific recommendations can be made as to the use of topical haemostats. The time to haemostasis is shorter when using topical haemostatic agents (fibrin sealant) but there is no significant difference in perioperative blood transfusion. There is no evidence of one individual topical sealant being superior to another.

*Recommendation grade:* moderate | median level of evidence: 1+ (range: 1++ to 2−)

**Expert panel comment**

Topical haemostatic agents, including matrix-based products, fibrin sealants, and their combination—known as carrier-bound fibrin sealants—are widely used in liver surgery for secondary haemostasis. Despite their popularity, the level of evidence supporting their effectiveness in reducing blood loss remains low.

A meta-analysis evaluating carrier-bound fibrin sealants in open and laparoscopic liver surgery, as well as transplantation, included RCTs, large retrospective cohort studies, and case–control studies^163^. While time to haemostasias was shorter in the carrier-bound fibrin sealant group, the use of these agents did not significantly affect the risk of intraoperative blood transfusion, postoperative fluid collections, or bile leakage.

Additionally, a network meta-analysis of 20 RCTs comparing different topical haemostatic agents reported that fibrin glue and fibrin patches were the most effective in achieving haemostasis at both 4 and 10 min^164^. No significant differences were however observed among agents in terms of overall blood loss, transfusion requirements, bile leak, postoperative complications, reoperation rates, or mortality.

Overall, while certain agents may improve time to haemostasis, their routine use does not appear to influence key perioperative outcomes in liver surgery^163^.

*Q6 Does minimally invasive surgery (MIS) influence blood loss in patients undergoing hepatectomy?*

MIS does not reduce blood loss compared to open liver surgery for minor or major hepatectomy. MIS liver surgery may be associated with less blood loss in some conditions (that is HCC) based on non-randomized observations.

*Recommendation grade:* strong (minor/major hepatectomy), conditional (HCC) | median level of evidence: 1− (range: 1++ to 2−)

**Expert panel comment**

In selected patients, MIS may decrease perioperative blood loss compared to the open approach. This is however only found when looking at specific resections (that is HCC) and most prominently in left lateral resections and in uncontrolled data comparisons^165–167^. Neither the Orange III RCT^168^ nor the COMET RCT^169^ demonstrated a statistically significant difference in blood loss attributable to the surgical approach.

**Topic 5:** PHH

Subtopic	Recommendation	Recommendation grade	Median level of evidence
Q1: What is the role of preoperative strategies to reduce blood loss associated with liver resection (fat-reducing diet, nutritional changes, hypovolaemic phlebotomy)?	In patients who are overweight or obese, the implementation of a low-calorie, low-fat diet should be considered, provided that the surgical timeline permits adequate intervention. Preoperative management of MASLD should align with the recommendations of the EASL–EASD–EASO Clinical Practice Guidelines. In selected patients at high risk of significant intraoperative bleeding, preoperative hypovolaemic phlebotomy may be considered as a strategy to reduce transfusion requirements.	Strong	2−
Q2: What is the role of anaesthetic management to reduce blood loss in patients undergoing hepatectomy?	Intraoperative low CVP should be maintained until completion of parenchymal transection to reduce blood loss and transfusion requirements. No specific anaesthetic agent is recommended. TXA is not advised in liver resection for cancer as it does not decrease blood loss during liver resection. The role of ANH remains unclear.	Moderate to strong	2++
Q3: What is the role for intraoperative liver inflow occlusion in reducing intraoperative blood loss?	Inflow occlusion techniques such as the PM and HHO can reduce intraoperative blood loss and should be considered when major bleeding is anticipated. While intermittent Pringle occlusion is safe for up to 120 min, prolonged clamping may increase the risk of ischaemia-reperfusion injury, particularly in patients with underlying liver dysfunction.	Moderate	2+
Q4: Is there an influence of parenchymal transection technique on blood loss?	Meta-analyses of RCTs suggest that bipolar energy devices may reduce intraoperative blood loss during parenchymal transection. However, due to the wide range of available techniques, the limited number of direct comparisons, and methodological variability across studies, no specific recommendation can currently be made regarding the optimal transection technique.	Moderate	1+
Q5: What is the value of haemostatic agents to reduce blood loss in patients undergoing hepatectomy?	No specific recommendations can be made as to the use of topical haemostats. The time to haemostasis is shorter when using topical haemostatic agents (fibrin sealant) but there is no significant difference in perioperative blood transfusion. There is no evidence of one individual topical sealant being superior to another.	Moderate	1+
Q6: Does MIS influence blood loss in patients undergoing hepatectomy?	MIS does not reduce blood loss compared to open liver surgery for minor or major hepatectomy. MIS liver surgery may be associated with less blood loss in some conditions (that is HCC) based on non-randomized studies.	Strong (minor/major hepatectomy), conditional (HCC)	1−

PHH, post-hepatectomy haemorrhage; MASLD, metabolic dysfunction-associated steatotic liver disease; EASL, European Association for the Study of the Liver; EASD, European Association for the Study of Diabetes; EASO, European Association for the Study of Obesity; CVP, central venous pressure; TXA, tranexamic acid; ANH, acute normovolaemic haemodilution; PM, Pringle manoeuvre; HHO, hemihepatic occlusion; MIS, minimally invasive surgery; HCC, hepatocellular carcinoma.

## Topic 6: PHLF

*Q1 Is there a clinical relevance to subclassify PHLF by the main underlying pathomechanism?*

PHLF involves complex and heterogeneous pathophysiological mechanisms and classification as primary and secondary PHLF can be considered, because it impacts on preventive and therapeutic interventions and prognosis.

*Recommendation grade:* conditional | median level of evidence: 2+ (range: 1+ to 3)

**Expert panel comment**

Primary PHLF defines liver failure (increased international normalized ratio (INR) and bilirubin according to local laboratory normal value ranges) and clinical signs of hepatic encephalopathy that occurs within days after liver resection (usual time frame within 3 weeks)^170^. Primary PHLF includes ‘small-for-size’ PHLF, in which the liver remnant is insufficient to maintain essential hepatic function, and iatrogenic PHLF, in which surgical injury of vital structures or excessive blood loss prevent sufficient function of the liver remnant^170,171^. Secondary PHLF occurs after an initial partial stabilization/recovery of hepatic function (usual time frame within 3 months after surgery) and is often complicated/aggravated by infections or hepatic vasculature thrombosis^170,172^.

While postoperative outcome research has predominantly focused on the first 90 postoperative days, it should be noted that delayed hepatic decompensation after this period can occur, particularly if infectious complications arise. Recent evidence supports this concept, as impaired preoperative liver function and/or occurrence of non-PHLF postoperative hepatic dysfunction is associated with long-term survival after hepatic resection^173–175^. This is particularly important as treatment strategies might differ between PHLF types. For example, Molecular Adsorbent Recirculating System (MARS) achieved better results as therapy of primary PHLF^176^ (especially if used early), while aggressive infection management is key in avoiding secondary PHLF^170^. Further research is required to strengthen the evidence of subclassifying PHLF, particularly for defining delayed PHLF occurring after 90 days.

*Q2 Should patients suffering from PHLF be monitored beyond 90 days after hepatectomy?*

Primary and secondary PHLF usually occur within 90 days after surgery. Based on indirect evidence, PHLF monitoring beyond 90 days can be considered for patients who have recovered from PHLF independent of oncological follow-up.

*Recommendation grade:* conditional | median level of evidence: 4 (range: 2+ to 4)

**Expert panel comment**

Primary and secondary PHLF occur within 90 days after liver resection. Delayed PHLF after 90 days—after initial stabilization/recovery of liver function—may occur, but further research is required to strengthen evidence for its definition, prediction, and management recommendations.

Recent evidence suggests that preoperative liver function and/or the development of postoperative non-PHLF hepatic dysfunction represents a critical predictor for long-term survival after hepatic resection^173–175,177^. Delayed PHLF may account for a significant proportion of post-resection mortality within 1 year, but strong supporting evidence is still missing. Monitoring beyond 90 days can be considered for those patients who have recovered from PHLF, but also for high-risk patients, who should be defined as per recent consensus guidelines^15^.

*Q3 Which postoperative markers should be considered to monitor postoperative liver function recovery in patients at high risk of PHLF?*

The following parameters should be used to monitor liver function in patients at high risk of PHLF: bilirubin, INR, albumin, lactate, ammonia levels (in patients with signs of hepatic encephalopathy), AST, alanine aminotransferase (ALT), glucose, and temperature. Furthermore, infection monitoring by complete blood count (CBC) and CRP should be performed, as well as kidney function monitoring using creatinine.

*Recommendation grade:* strong | median level of evidence: 2+ (range: 2+ to 4)

**Expert panel comment**

After hepatic resection the following parameters should be used to monitor liver function in patients at high risk of PHLF: bilirubin, INR, albumin, ammonia levels (in patients with signs of hepatic encephalopathy), glucose, temperature, aminotransferases (AST/ALT), infection markers (leucocytes, procalcitonin, and CRP), lactate, and kidney function (creatinine).

If clinically indicated, radiological assessment of the liver perfusion (ultrasound or CT scan) can be helpful to identify vascular complications. In this context, high-risk patients should be defined as per recent consensus guidelines^15^.

*Q4 Is there a value for early postoperative markers to predict PHLF?*

Several biomarkers (for example lactate, phosphate, platelets) have been reported to allow for early postoperative identification of PHLF and their measurement can be considered in high-risk patients after hepatic resection to initiate supportive treatment early. There are no comparative studies and future research is needed to identify the optimal biomarker.

*Recommendation grade:* conditional | median level of evidence: 2+ (range: 2+ to 4)

**Expert panel comment**

The postoperative parameters reported to be useful for early identification of PHLF patients include lactate levels^178–180^, fibrosis-4 (FIB-4) score^181^, platelets^182^, ICG clearance (intra- and postoperative)^183,184^, phosphate^185,186^, AT III and CRP^187^, scintigraphic parameters^188,189^, thrombopoietin^190^, and intrahepatic neutrophil accumulation^191^. At this point in time direct comparisons of these markers are missing. Accordingly, no recommendation can be made regarding which parameter is superior. Given that supportive treatments (particularly for primary PHLF) should be initiated early^176^, biomarker identification and comparison should become a key area of future research. Moreover, these markers will be instrumental for establishing and eventually initiating therapeutic interventions in the future^192,193^. In this context, high-risk patients should be defined as per recent consensus guidelines^15^.

*Q5 Which treatments are available to support hepatic regeneration in PHLF?*

Currently, no specific therapeutic interventions are available or approved to promote liver regeneration and avoid or treat progression of PHLF.

*Recommendation grade:* strong | median level of evidence: 2+ (range: 1+ to 4)

**Expert panel comment**

Supportive care and aggressive management of potential causes of PHLF are recommended. While several treatments have been explored, no specific therapy is currently available. Antioxidant drugs, such as *N*-acetylcysteine, have shown mixed results for treatment of PHLF and their use is controversial^194,195^. Bioartificial devices, mesenchymal stem cells, and new molecular targets are currently being explored in patients at risk of or already suffering from PHLF. Portal pressure modulators like terlipressin and somatostatin have demonstrated mixed results as well. Surgical modulation of portal pressure by splenectomy or splenic artery ligation has also been implicated in PHLF reduction^196^. MARS and plasma exchange therapies have also been evaluated with inconsistent results^197–199^. Liver transplantation is an effective treatment for fulminant PHLF but is seldomly possible given patient factors like oncological diagnosis or infections^200–202^. Preoperative or early postoperative identification of high-risk patients remains key to enabling timely initiation of supportive treatments.

**Topic 6:** PHLF

Subtopic	Recommendation	Recommendation grade	Median level of evidence
Q1: Is there a clinical relevance to subclassify PHLF by the main underlying pathomechanism?	PHLF involves complex and heterogeneous pathophysiological mechanisms and classification as primary and secondary PHLF can be considered, because it impacts on preventive and therapeutic interventions and prognosis.	Conditional	2+
Q2: Should patients suffering from PHLF be monitored beyond 90 days after hepatectomy?	Primary and secondary PHLF usually occur within 90 days after surgery. Based on indirect evidence, PHLF monitoring beyond 90 days can be considered for patients who have recovered from PHLF independent of oncological follow-up.	Conditional	4
Q3: Which postoperative markers should be considered to monitor postoperative liver function recovery in patients at high risk of PHLF?	The following parameters should be used to monitor liver function in patients at high risk of PHLF: bilirubin, INR, albumin, lactate, ammonia levels (in patients with signs of hepatic encephalopathy), AST, ALT, glucose, and temperature. Furthermore, infection monitoring by CBC and CRP should be performed, as well as kidney function monitoring using creatinine.	Strong	2+
Q4: Is there a value for early postoperative markers to predict PHLF?	Several biomarkers (for example lactate, phosphate, platelets) have been reported to allow for early postoperative identification of PHLF and their measurement can be considered in high-risk patients after hepatic resection to initiate supportive treatment early. There are no comparative studies and future research is needed to identify the optimal biomarker.	Conditional	2+
Q5: Which treatments are available to support hepatic regeneration in PHLF?	Currently, no specific therapeutic interventions are available or approved to promote liver regeneration and avoid or treat progression of PHLF.	Strong	2+

PHLF, post-hepatectomy liver failure; INR, international normalized ratio; AST, aspartate aminotransferase; ALT, alanine aminotransferase; CBC, complete blood count; CRP, C-reactive protein.

## Discussion

This consensus paper builds upon and complements the E-AHPBA–ESSO–European Society for Surgical Research (ESSR) Innsbruck consensus guidelines on preoperative liver function assessment published in *BJS* in 2023^15^, providing a continuum of guidance from preoperative evaluation to postoperative care. Developed by a multidisciplinary expert panel, it aims to reduce practice variability and improve patient outcomes by synthesizing the best available evidence with the opinions and clinical knowledge of experts in the field. It provides a structured framework for peri- and postoperative management in patients undergoing liver resection, addressing six critical domains: thromboprophylaxis, antibiotics, prehabilitation, bile leak, bleeding, and liver failure. A total of 32 statements were generated.

Even though the review of the literature revealed that most of the available evidence informing these recommendations is limited in both quantity and quality, the expert panel has identified key practices that, if implemented, are believed to support improved outcomes in peri- and postoperative care following liver surgery.

This consensus recommendation is subject to several limitations. These include heterogeneity of definitions, particularly regarding perioperative complications but also regarding liver surgery procedures and indications. The variability in the application of interventions such as prehabilitation, thromboprophylaxis, and bile leak management limit generalizability. Several recommendations also rely on advanced diagnostics and therapeutics not universally available, challenging implementation across diverse healthcare settings. Many of the recommendations have been based on low- to moderate-quality evidence, often derived from retrospective analyses, institutional protocols, or expert opinion, due to the lack of high-quality RCTs in key areas. As with many expert consensus processes, the strength of the recommendations reflects both the current evidence base and clinical judgment.

It is necessary to consider the demographic and geographical composition, as well as the clinical specialty, of the involved experts. Most of the faculty comes from high-income countries and were engaged in high-volume hospitals with specialized hepatobiliary surgery units. It is therefore evident that the clinical experience and available resources of the aforementioned units may not be directly transferable to low- or medium-volume surgical units or to healthcare settings characterized by resource constraints. Consequently, the implementation of any recommendation outlined in this consensus statement is contingent upon the specific requirements, experience, and available resources of the respective institutions or countries.

The consensus threshold of 70% allows the potential inclusion of statements with limited evidence. Further limitations arise from the methodology applied. Incorporating a modified Delphi approach and expert opinions carries some risk of bias in the interpretation of the literature review, especially as members of the consensus group were involved in many publications retrieved in the literature search. Involvement of a neutral validation committee and full transparency through declaration of conflict of interest for each co-author addressed this potential limitation. Finally, long-term outcome data are sparse, particularly in relation to late postoperative complications, underscoring the need for prospective, multicentre studies to validate and refine these recommendations.

Clinicians are encouraged to adapt these consensus recommendations according to institutional resources, local expertise, and patient populations. The recommendations are designed to align with other principles, such as Enhanced Recovery After Surgery (ERAS)^6^ and EASL–EASD–EASO Clinical Practice Guidelines^149^. They are also implementable within both academic and community practice environments. Given the level of available evidence, the current rate of innovation in perioperative management of surgical patients, including those undergoing liver surgery, and the ongoing trials, the authors estimate that these recommendations will at least remain relevant for approximately 5 years.

To strengthen future versions of these recommendations and resolve ongoing uncertainties, several evidence gaps on perioperative management of patients undergoing liver resections have been identified throughout this consensus development process. These clinically relevant areas represent future research topics that could be addressed in a common, multicentric effort with the aim to improve clinical outcomes and standardize the perioperative management of patients undergoing complex liver resections. Equally, some topics were identified where additional research may yield limited clinical benefit and that the authors define as ‘futile’ since enough good evidence has been achieved and no future efforts should be invested in those areas (*Table 2*).

**Table 2:** Areas where future research may be futile

Area	Rationale
Routine use of intraoperative drains	Multiple RCTs have shown no benefit of abdominal drains in standard hepatectomies. Enhanced recovery protocols strongly advocate against routine use of drains as they may hinder early mobilization.
MIS and blood loss	Recent RCTs provide high level of evidence that MIS does not result in significantly lower blood loss as compared to open surgery.

MIS, minimally invasive surgery.

The present consensus paper has been developed for the purpose of supporting clinicians in the delivery of safe, evidence-informed, and standardized care across the perioperative course of liver resection. The text addresses fundamental clinical dilemmas, with the objective of providing clear guidance, thus making it a practical reference for surgical and multidisciplinary teams. It is imperative that ongoing research collaboration, implementation monitoring, and periodic updates are maintained to ensure the continued relevance of the programme and to enhance the outcomes for patients undergoing liver resection.

## Supplementary Material

znaf272_Supplementary_Data

## Data Availability

Not applicable.
