# Supplementary material for: The E-AHPBA—ESSO—Innsbruck consensus recommendations on peri- and postoperative management following liver resection
Source: Br J Surg. 2025 Dec 30;113(1):znaf272. doi: 10.1093/bjs/znaf272 (PMC12750329; doi:10.1093/bjs/znaf272)
Supplement: znaf272_Supplementary_Data [file znaf272_supplementary_data.docx]

**Supplementary material**

**The E-AHPBA – ESSO – Innsbruck Consensus Guidelines on
Peri- and Postoperative Management Following Liver Resection**

*Maier Eva^1^*, Stättner Stefan^2^*, Carrión-Álvarez Lucia^3^, Di Martino Marcello^4^, Olthof Pim^5^, Primavesi Florian^1^, Socharova Dana^6^, van Laarhoven Stijn^7^, Balakrishnan Anita^8^, Breitkopf Robert^9^, Bruis Carlijn^10^, Cipriani Federica^11^, Erdmann Joris^12^, Frampton Adam^13^, Fuks David^14^, Gilg Stefan^15^, Kielaite-Gulla Aiste^16^, Lancellotti Francesco^17^, Margreiter Christian^18^, Melloul Emmanuel^14^, Oberkofler Christian^19^, Petritsch Stefan^20^, Raab Helmut^9^, Rahbari Nuh N.^21,^ Rappold Daniela^22^, Reiberger Thomas^23^, Ruzzenente Andrea^24^, Sallinen Ville^25^, Schäfer Benedikt^26^, Schnitzbauer Andreas^27^, Serrablo Antonio^28^, Soreide Kjetil^29^, Sparrelid Ernesto^15^, Starlinger Patrick^30^, Stavrou Gregor^31^, Tinguely Pascale^32^, Aldrighetti Luca^11^, Dasari Bobby^33^, Donadon Matteo^4^, Dopazo Christina^34^, Grünberger Thomas^35^, Jonas Eduard^36^, Malik Hassan^37^, Viganó Luca^38^, Siriwardena Ajith K ^39**^, Maglione Manuel^1**^*

* contributed equally

** shared corresponding authorship

**Supplementary Materials - Index**

| 1. **Supplementary Methods** | |  | |
| --- | --- | --- | --- |
| Systematic Literature Review | | *page 3* | |
|  | |  | |
| 1. **Supplementary Tables** | |  | |
| Table S1. Inclusion and exclusion criteria | | *page 4* | |
| Table S2. Quality of Evidence  Table S3. Evidence Levels and Recommendation | | *page 4*  *page 5* | |
|  | |  | |
| 1. **Supplementary Results** | |  | |
| Literature Review | | *page 6* | |
|  | |  | |
| 1. **Supplementary Figures** | |  | |
| Quality Control by Validation Committee | | *page 25* | |
|  | |  | |
|  | |  | |
|  | |  | |
|  | |  | |

1. **Supplementary Methods**

*Systematic Literature Review (performed by working groups)*

Literature review was performed including the following databases:

- Cochrane Library
  - -  for reviews: the Cochrane Library
  - -  for RCTs: Cochrane Central Register of Controlled Trials
- PUBMED / Medline
- Embase
- Web of sciences

The following additional resources were also considered:

- Internet sites relevant to the topic (including patient organizations)
- WHO International Clinical Trials Registry Platform
- References or citations associated with the publications retrieved through the search

Keywords for literature search

Each working group performed the literature search according to several pre-fixed keywords combined with individual search terms for each topic (see below for each group):

Pre-fixed keyword strategy to be included in every search:

(english[Language] AND ("2010/01/01"[Date - Publication]: "2024/03/26"[Date - Publication]) AND ("'hepatectomy'/exp OR ('metastasis resection'/exp AND 'liver'/exp) OR (((liver OR hepat*) NEAR/3 (surgery OR surgeries OR resection* OR lobectom* OR metastasectom) OR hepatectom OR hemihepatectom OR trisectionectom)

NOT (transplant, transplantation, donor, graft, grafts, mice, mouse, murine, pig, pigs, porcine, rat, rats, animal, animals, conference abstract, conference review, editorial, preprint)

Combined with individual keywords for each topic (e.g. in case of bile leakage):

AND (bile leakage OR bilioenteric anastomosis OR bilioenteric fistula OR postoperative complication AND bile leak OR bile fistula OR bilioenteric anastomosis OR bilio-enteric anastomosis OR anastomosis leakage AND bile OR biliar OR bilio OR post hepatectom OR postoperat OR post operat OR postsurg OR post surg OR after hepatectomy OR after partial hepatectomy OR after operation OR after surgery OR following hepatectomy OR following partial hepatectomy OR following operation OR following surgery etc.

1. **Supplementary Tables**

| **Table S1. Inclusion and exclusion criteria for the literature research performed for this guideline** | |
| --- | --- |
| ***Inclusion criteria*** | |
| Publication date | 01/2010 – 03/2024 (standard search timeframe) |
| Extended publication date | Before 01/2010 and after 04/2024 for important references derived from other publications or in case of older / very recent landmark papers |
| Language and type of article | Published full-text articles in English language |
| Types of publications | - Systematic reviews - Meta-Analysis - Randomized controlled trials - Prospective studies - Expert Consensus - Retrospective studies (only be considered if better data are not available) |
| ***Exclusion criteria*** | |
| Types of publications | - Animal studies without human data - Basic science studies without clinical data - Publications without English abstract or with no full paper available (congress abstracts etc.) - Case reports / studies with less than 10 cases - Duplicate data publication |

| **Table S2. Quality of Evidence and Corresponding Evidence Level (SIGN Methodology)** | |
| --- | --- |
| **Quality of Evidence** | **Evidence level** |
| High-quality meta-analyses, systematic reviews of RCTs, or RCTs with a very low risk of bias. | 1++ |
| Well conducted meta-analyses, systematic reviews, or RCTs with a low risk of bias. | 1+ |
| Meta-analyses, systematic reviews, or RCTs with a high risk of bias. | 1- |
| High-quality systematic reviews of case-control or cohort studies.  High-quality case-control or cohort studies with a very low risk of confounding or bias and a high probability the relationship is causal. | 2++ |
| Well-conducted case-control or cohort studies with a low risk of confounding or bias and a moderate probability that the relationship is causal. | 2+ |
| Case-control or cohort studies with a high risk of confounding or bias and a significant risk that the relationship is not causal. | 2- |
| Nonanalytic studies, for example, case reports, case series. | 3 |
| Expert opinion. | 4 |

| **Table S3. Evidence Levels and Corresponding Forms of Recommendations (SIGN Methodology)** | |
| --- | --- |
| **Judgment** | **Form of Recommendation** |
| Undesirable consequences clearly outweigh desirable consequences | Strong recommendation against |
| Desirable consequences clearly outweigh undesirable consequences | Strong recommendation for |
| Undesirable consequences probably outweigh desirable consequences | Conditional recommendation against |
| Desirable consequences probably outweigh undesirable consequences | Conditional recommendation for |
| Strong recommendations are more likely associated to high quality evidence (1++/1+/1-/2++); lower quality evidence (2+/2-/3/4) are associated with other forms of recommendations. | |

1. **Supplementary Results**

*Literature Review*

Group 1: Perioperative thromboprophylaxis

Specific additional keywords:

TS=((peri-operat* OR perioperat* OR "during hepatectomy" OR "during partial hepatectomy" OR "during operation" OR "during surgery" OR prophylaxis OR prophylactic) NEAR/6 (anticoag* OR antithromb* OR antiplatelet OR thromboembol* OR thrombos* OR heparin* OR warfarin OR "vitamin k antagonist*" OR "factor Xa inhibitor*" OR "factor 10a inhibitor*" OR "direct thrombin inhibitor*" OR aldocumar OR coumadin* OR marevan OR tedicumar OR aspirin OR 4-hydroxycoumarins OR Abiciximab OR Acenocoumarol OR Ancrod OR Becaplermin OR "beta 2-glycoprotein I" OR "citric acid" OR dabigatran OR dalteparin* OR "dermatan sulfate" OR dextrans OR dicumarol OR "edetic acid" OR enoxaparin* OR "ethyl biscoumacetate" OR "fibrin* degradation product*" OR fondaparinux OR Gabexate OR Hirudin* OR bivalirudin OR nadroparin* OR phenindione OR phenprocoumon OR "protein C" OR "protein S" OR Rivaroxaban OR Pradaxa OR Xarelto OR apixaban OR Eliquis OR edoxaban OR Savaysa OR betrixaban OR "sodium citrate" OR Tinzaparin* OR argatroban)) OR TS=((peri-operat* OR perioperat* OR "during hepatectomy" OR "during partial hepatectomy" OR "during operation" OR "during surgery") AND thromboprophyl*)

Number of initially retrieved search results: 383

Excluded studies after screening by working group and steering committee: 368

Final number of studies: 16

| **Q1: When should perioperative thromboprophylaxis be commenced in liver surgery? (n=2)** | | | | | | |
| --- | --- | --- | --- | --- | --- | --- |
| **PMID** | **First author** | **Year** | **Journal** | **Type** | **Title** | **LOE** |
| **33223433** | Ainoa | 2021 | HBP (Oxford) | Retrospective study | Pre- vs. postoperative initiation of thromboprophylaxis in liver surgery | 2+ |
| **25435300** | Doughtie | 2014 | Am J Surg | Retrospective study | Preoperative dosing of low-molecular-weight heparin in hepatopancreatobiliary surgery | 2+ |

| **Q2: What is the optimal duration of perioperative thromboprophylaxis in liver surgery? (n=4)** | | | | | | |
| --- | --- | --- | --- | --- | --- | --- |
| **PMID** | **First author** | **Year** | **Journal** | **Type** | **Title** | **LOE** |
| 24888461 | Weiss | 2014 | HBP (Oxford) | Retrospective study | Venous thromboembolic prophylaxis after a hepatic resection: patterns of care among liver surgeons | 5 |
| 34591148 | Hanna | 2022 | World J Surg | Retrospective study | Incidence and Risk Factors of Venous Thromboembolism Following Hepatectomy for Colorectal Metastases: A Population-Based Retrospective Cohort Study | 2+ |
| 28846822 | Kim | 2017 | J Thromb Haemost | Retrospective study | Extended pharmacologic thromboprophylaxis in oncologic liver surgery is safe and effective | 2- |
| 29472105 | Beal | 2018 | HBP (Oxford) | Retrospective study | Identification of patients at high risk for post-discharge venous thromboembolism after hepato-pancreato-biliary surgery: which patients benefit from extended thromboprophylaxis? | 2- |

| **Q3: What anticoagulation should be used when performing hepatectomy with vascular resection and reconstruction? (n= 10)** | | | | | | |
| --- | --- | --- | --- | --- | --- | --- |
| **PMID** | **First author** | **Year** | **Journal** | **Type** | **Title** | **LOE** |
| 30841029 | Agarwal | 2019 | Korean J Anesthesiol | Narrative review | Enhanced recovery after surgery in liver resection: current concepts and controversies | 2- |
| 33223433 | Ainoa | 2021 | HBP (Oxford) | Retrospective study | Pre- vs. postoperative initiation of thromboprophylaxis in liver surgery | 2+ |
| 29472105 | Beal | 2018 | HBP (Oxford) | Retrospective study | Identification of patients at high risk for post-discharge venous thromboembolism after hepato-pancreato-biliary surgery: which patients benefit from extended thromboprophylaxis? | 2+ |
| 25435300 | Doughtie | 2014 | Am J Surg | Retrospective study | Preoperative dosing of low-molecular-weight heparin in hepatopancreatobiliary surgery | 2+ |
| 31185468 | Eguchi | 2020 | Digestive surgery | Prospective phase I study | A Prospective, Multi-Center Phase I Study of Postoperative Enoxaparin Treatment in Patients Undergoing Curative Hepatobiliary-Pancreatic Surgery for Malignancies | 1+ |
| 24337986 | Ejaz | 2014 | Journal of Gastrointestinal Surgery | Retrospective study | Defining incidence and risk factors of venous thromboemolism after hepatectomy | 2+ |
| 24687760 | Hayashi | 2014 | Surgery Today | Retrospective study | Safety of postoperative thromboprophylaxis after major hepatobiliary–pancreatic surgery in Japanese patients | 2- |
| 35881311 | Karunakaran | 2022 | Langenbecks Arch Surg | Meta-analysis | Post-hepatectomy venous thromboembolism: a systematic review with meta-analysis exploring the role of pharmacological thromboprophylaxis | 2- |
| 28069106 | Miyazaki | 2017 | Am J Surg | Retrospective study | Portal vein thrombosis after reconstruction in 270 consecutive patients with portal vein resections in hepatopancreatobiliary (HPB) surgery | 2+ |
| 28012034 | Molina | 2016 | Langenbecks Arch Surg | Retrospective study | Surgical treatment of perihilar cholangiocarcinoma: early results of en bloc portal vein resection | 2+ |

| **Q4: Is there a role for defining early and late portal vein thrombosis after liver resection? (n=0)**  **No papers defining early and late portal vein thrombosis in the context of liver surgery (papers screened n= 384)** |
| --- |

| **Q5: Are there differences in the treatment of early and late portal vein thrombosis after liver resection? (n=0)**  **No evidence on which treatment strategy could be recommended for postoperative early and late portal vein thrombosis (papers screened n= 384)** |
| --- |

Group 2: Perioperative antibiotics

Specific additional keywords:

TS=((liver OR hepat*) NEAR/3 (surgery OR surgeries OR resection* OR lobectom* OR metastasectom*)) OR TS=(hepatectom* OR hemihepatectom* OR trisectionectom*) OR TS=(cholecystostom* OR portoenterostom* OR choledochostom* OR choledochojejunostom* OR hepatojejunostom* OR "hepato biliopancreatic*" OR (HBP NEAR/2 (surgery OR surgeries))) OR TS=(biliary NEAR/2 tract NEAR/3 (drain* OR surgery OR surgeries OR surgical OR procedur*)) OR TS=(bile NEAR/2 duct NEAR/3 (drain* OR surgery OR surgeries OR surgical OR procedur*))

Number of initially retrieved search results: 1176

Excluded studies after screening by working group and steering committee: 86

Final number of studies: 51

| **Q1: Which antibiotics should be used as standard prophylaxis for liver surgery? (n=20)** | | | | | | |
| --- | --- | --- | --- | --- | --- | --- |
| **PMID** | **First author** | **Year** | **Journal** | **Type** | **Title** | **LOE** |
| 22071832 | Gurusamy | 2011 | The Cochrane database of systematic reviews | Systematic Review | Methods of decreasing infection to improve outcomes after liver resections | 1++ |
| 26715500 | Zhou | 2016 | Dig Dis Sci | Observational Study | Preoperative Antibiotic Prophylaxis Does Not Reduce the Risk of Postoperative Infectious Complications in Patients Undergoing Elective Hepatectomy | 2++ |
| 31261586 | Guo | 2019 | Medicine | Network Meta-analysis | Evaluation of different antibiotic prophylaxis strategies for hepatectomy: A network meta-analysis | 1++ |
| 23706259 | Hirokawa | 2013 | Am J Surg | RCT | Evaluation of postoperative antibiotic prophylaxis after liver resection: a randomized controlled trial | 1+ |
| 32398496 | Liew | 2021 | Eur J Gastroenterol Hepatol | Observational Study | Reducing prophylactic antibiotics use in laparoscopic cholecystectomy and liver resections | 2+ |
| 35203797 | Steccanella | 2022 | Antibiotics | Systematic Review | Antibiotic Prophylaxis for Hepato-Biliopancreatic Surgery-A Systematic Review | 1++ |
| 26573175 | Matsumura | 2016 | World J Surg | Observational Study | High Rate of Organ/Space Surgical Site Infection After Hepatectomy with Preexisting Bilioenteric Anastomosis | 2+ |
| 30246017 | Tang | 2018 | BioMed research international | Propensity Score Study | Single Dose Based Ertapenem Prophylaxis Reduces Surgical Site Infection after Selective Hepatectomy of Hepatocellular Carcinoma: A Propensity Score Matching Study | 2+ |
| 38078132 | Liakina | 2023 | World J Clin Cases | Narrative Review | Antibiotic resistance in patients with liver cirrhosis: Prevalence and current approach to tackle | 2+ |
| 23557410 | Ceppa | 2013 | HBP (Oxford) | Observational Study | Reducing surgical site infections in hepatopancreatobiliary surgery | 2+ |
|  | DaVee | 2017 | Gastrointestinal endoscopy | Observational Study | Malignant intrahepatic biliary obstructions: do antibiotics prevent cholangitis in patients with incomplete biliary drainage? | 2+ |
| 30631393 | Dirchwolf | 2018 | World J Hepatol | Review | Unresolved issues in the prophylaxis of bacterial infections in patients with cirrhosis | 2+ |
| 27759623 | Sugawara | 2018 | Ann Surg | RCT | Duration of Antimicrobial Prophylaxis in Patients Undergoing Major Hepatectomy with Extrahepatic Bile Duct Resection: A Randomized Controlled Trial | 1+ |
| 29380135 | Ishioka | 2018 | Surgery Today | Observational Study | Significance of bacterial culturing of prophylactic drainage fluid in the early postoperative period after liver resection for predicting the development of surgical site infections | 2+ |
| 25830815 | Isik | 2015 | Surgical infections | Observational Study | Factors Affecting Surgical Site Infection Rates in Hepatobiliary Surgery | 2+ |
| 28371248 | Okamura | 2017 | Journal of hepato-biliary-pancreatic sciences | RCT | Randomized controlled trial of perioperative antimicrobial therapy based on the results of preoperative bile cultures in patients undergoing biliary reconstruction | 1+ |
| 23327981 | Bratzler | 2013 | Am J Health Syst Pharm | Guideline | Clinical practice guidelines for antimicrobial prophylaxis in surgery | 1++ |
| 31983392 | Badia | 2020 | Cir Esp | Position Statement | Surgical site infection prevention measures in General Surgery: Position statement by the Surgical Infections Division of the Spanish Association of Surgery | 2+ |
| 33536484 | Bednarsch | 2021 | Scientific reports | Observational Study | Bacterial bile duct colonization in perihilar cholangiocarcinoma and its clinical significance | 2+ |
| 30064539 | Stack | 2018 | Infection control and hospital epidemiology | Review | Perioperative antimicrobial prophylaxis and prevention of hepatobiliary surgical site infections | 2+ |

| **Q2: Which patients should be defined as high-risk for developing clinically relevant SSI following hepatectomy? (n=25)** | | | | | | | |
| --- | --- | --- | --- | --- | --- | --- | --- |
| **PMID** | **First author** | **Year** | **Journal** | **Type** | **Title** | **LOE** |  |
| 28371248 | Okamura | 2017 | J Hepatobiliary Pancreat Sci | RCT | Randomized controlled trial of perioperative antimicrobial therapy based on the results of preoperative bile cultures in patients undergoing biliary reconstruction | 1+ |  |
| 30064539 | Bednarsch | 2021 | Scientific reports | Observational Study | Bacterial bile duct colonization in perihilar cholangiocarcinoma and its clinical significance | 2+ |  |
| 31229489 | Chacon | 2019 | HBP (Oxford) | Observational Study | Effect of operative duration on infectious complications and mortality following hepatectomy | 2+ |  |
|  | Chambers | 2022 | AIMS BIOENGINEERING | Systematic Review | A systematic review on the incidence and risk factors of surgical site infections following hepatopancreatobiliary (HPB) surgery | 1++ |  |
| 26333471 | Dong | 2015 | HBP (Oxford) | Observational Study | Prior inpatient admission increases the risk of post-operative infection in hepatobiliary and pancreatic surgery | 2+ |  |
| 26573175 | Matsumura | 2016 | World J Surg | Observational Study | High Rate of Organ/Space Surgical Site Infection After Hepatectomy with Preexisting Bilioenteric Anastomosis | 2+ |  |
| 24519844 | Nakayama | 2014 | J Hepatobiliary Pancreat Sci | RCT | Subcutaneous drainage to prevent wound infection in liver resection: a randomized controlled trial | 1+ |  |
| 35618490 | Ruzzenente | 2022 | Surgery | Observational Study | Infectious complications after surgery for perihilar cholangiocarcinoma: A single Western center experience | 2+ |  |
| 31646746 | Shen | 2020 | Int Wound J | Observational Study | Clinical prediction score for superficial surgical site infections: Real-life data from a retrospective single-centre analysis of 812 hepatectomies | 2+ |  |
| 25830815 | Isik | 2015 | Surgical infections | Observational Study | Factors Affecting Surgical Site Infection Rates in Hepatobiliary Surgery | 2+ |  |
| 25561190 | Kokudo | 2015 | World J Surg | Observational Study | Risk factors for incisional and organ space surgical site infections after liver resection are different | 2+ |  |
| 30001844 | Takahashi | 2018 | Journal of Infection and Chemotherapy | Observational Study | Risk factors for surgical site infection after major hepatobiliary and pancreatic surgery | 2+ |  |
| 28445320 | Tang | 2017 | Medicine (Baltimore) | Observational Study | Risk factors and long-term outcome for postoperative intra-abdominal infection after hepatectomy for hepatocellular carcinoma | 2+ |  |
| 20676699 | Uchiyama | 2011 | J Hepatobiliary Pancreat Sci | Observational Study | Risk factors for postoperative infectious complications after hepatectomy | 2+ |  |
| 24521601 | Yang | 2014 | Infect Control Hosp Epidemiol | Observational Study | Risk factors of surgical site infection after hepatic resection | 2+ |  |
| 26573175 | Matsumura | 2016 | World J Surg | Observational Study | High Rate of Organ/Space Surgical Site Infection After Hepatectomy with Preexisting Bilioenteric Anastomosis | 2+ |  |
| 32785844 | Morikawa | 2021 | Surg Today | Observational Study | Liver resections in patients with prior bilioenteric anastomosis are predisposed to develop organ/space surgical site infections and biliary leakage: results from a propensity score matching analysis | 2+ |  |
| 22273719 | Sadamori | 2013 | J Hepatobiliary Pancreat Sci | Observational Study | Risk factors for organ/space surgical site infection after hepatectomy for hepatocellular carcinoma in 359 recent cases | 2+ |  |
| 22086698 | Kaibori | 2011 | Hepato-gastroenterology | Observational Study | Postoperative infectious and non-infectious complications after hepatectomy for hepatocellular carcinoma | 2+ |  |
| 30243876 | Martin | 2018 | HBPD INT | Observational Study | Impact of postoperative intravenous fluid administration on complications following elective hepato-pancreato-biliary surgery | 2+ |  |
| 28640107 | Li | 2017 | Medicine (Baltimore) | Observational Study | A nomogram prediction of postoperative surgical site infections in patients with perihilar cholangiocarcinoma | 2+ |  |
| 22273719 | Sadamori | 2013 | J Hepatobiliary Pancreat Sci | Observational Study | Risk factors for organ/space surgical site infection after hepatectomy for hepatocellular carcinoma in 359 recent cases | 2+ |  |
| 36622470 | Yasuda | 2023 | Langenbecks Arch Surg | Observational Study | Pre- and postoperative C-reactive protein as a risk factor of organ/space surgical site infection after hepatectomy | 2+ |  |
| 28637037 | Shirata | 2018 | Digestive surgery | Observational Study | Surgical Site Infection after Hepatectomy for Hepatocellular Carcinoma | 2+ |  |
| 35093901 | Yusa | 2022 | Anticancer research | Observational Study | Efficacy of Ring Drape and Unused Sterile Instruments for Incisional SSI After Hepatectomy | 2+ |  |

| **Q3: Which patients are likely to benefit from extended antibiotic prophylaxis? (n=30)** | | | | | | |
| --- | --- | --- | --- | --- | --- | --- |
| **PMID** | **First author** | **Year** | **Journal** | **Type** | **Title** | **LOE** |
| 23706259 | Hirokawa | 2013 | Am J Surg | RCT | Evaluation of postoperative antibiotic prophylaxis after liver resection: a randomized controlled trial | 1+ |
| 22071832 | Gurusamy | 2011 | The Cochrane database of systematic reviews | Systematic Review | Methods of decreasing infection to improve outcomes after liver resections | 1++ |
|  | Chambers | 2022 | AIMS BIOENGINEERING | Systematic Review | A systematic review on the incidence and risk factors of surgical site infections following hepatopancreatobiliary (HPB) surgery | 1++ |
| 27759623 | Sugawara | 2018 | Annals of surgery | RCT | Duration of Antimicrobial Prophylaxis in Patients Undergoing Major Hepatectomy With Extrahepatic Bile Duct Resection: A Randomized Controlled Trial | 1+ |
| 26715500 | Zhou | 2016 | Digestive diseases and sciences | Observational Study | Preoperative Antibiotic Prophylaxis Does Not Reduce the Risk of Postoperative Infectious Complications in Patients Undergoing Elective Hepatectomy | 2+ |
| [31261586](https://pubmed.ncbi.nlm.nih.gov/31261586/) | Guo | 2019 | Medicine | Network Meta-analysis | Evaluation of different antibiotic prophylaxis strategies for hepatectomy: A network meta-analysis | 1++ |
| 34623761 | Gupta | 2022 | J Hepatobiliary Pancreat Sci | RCT | Randomized control trial on perioperative antibiotic prophylaxis in live liver donors: Are three doses enough? | 1+ |
| 33398563 | Matsukuma . | 2021 | Surgical endoscopy | Observational Study | Laparoscopic resection reduces superficial surgical site infection in liver surgery | 2+ |
| [35625294](https://pubmed.ncbi.nlm.nih.gov/35625294/) | Murtha-Lemekhova . | 2022 | Antibiotics | Systematic Review | Routine Postoperative Antibiotic Prophylaxis Offers No Benefit after Hepatectomy-A Systematic Review and Meta-Analysis | 1++ |
| 24509400 | Nanashima | 2014 | International journal of surgery | Observational Study | Associated factors with surgical site infections after hepatectomy: predictions and countermeasures by a retrospective cohort study | 2+ |
| 28371248 | Okamura | 2017 | J Hepatobiliary Pancreat Sci | RCT | Randomized controlled trial of perioperative antimicrobial therapy based on the results of preoperative bile cultures in patients undergoing biliary reconstruction | 1+ |
| 27906606 | Sakoda | 2017 | Surgical infections | Observational Study | Influence of a Shorter Duration of Post-Operative Antibiotic Prophylaxis on Infectious Complications in Patients Undergoing Elective Liver Resection | 2+ |
| [31549010](https://pubmed.ncbi.nlm.nih.gov/31549010/) | Shinkawa | 2019 | Annals of gastroenterological surgery | Observational Study | Giving short-term prophylactic antibiotics in patients undergoing open and laparoscopic hepatic resection | 2+ |
| 30064539 | Stack | 2018 | Infection control and hospital epidemiology | Review | Perioperative antimicrobial prophylaxis and prevention of hepatobiliary surgical site infections | 2+ |
| [35203797](https://pubmed.ncbi.nlm.nih.gov/35203797/) | Steccanella | 2022 | Antibiotics | Systematic Review | Antibiotic Prophylaxis for Hepato-Biliopancreatic Surgery-A Systematic Review | 1++ |
| 31030266 | Takayama | 2019 | Surgery Today | RCT | Antimicrobial prophylaxis for 1 day versus 3 days in liver cancer surgery: a randomized controlled non-inferiority trial | 1+ |
| 30246017 | Tang . | 2018 | BioMed research international | Propensity Score Matching Study | Single Dose Based Ertapenem Prophylaxis Reduces Surgical Site Infection after Selective Hepatectomy of Hepatocellular Carcinoma: A Propensity Score Matching Study | 2+ |
| 30064539 | Bednarsch | 2021 | Scientific reports | Observational Study | Bacterial bile duct colonization in perihilar cholangiocarcinoma and its clinical significance | 2+ |
| 31229489 | Chacon | 2019 | HBP (Oxford) | Observational Study | Effect of operative duration on infectious complications and mortality following hepatectomy | 2+ |
| 34732438 | Furukawa | 2021 | Anticancer research | Observational Study | Risk Factors and Overcoming Strategies of Surgical Site Infection After Hepatectomy for Colorectal Liver Metastases | 2+ |
| 29380135 | Ishioka | 2018 | Surgery Today | Observational Study | Significance of bacterial culturing of prophylactic drainage fluid in the early postoperative period after liver resection for predicting the development of surgical site infections | 2+ |
| 25830815 | Isik | 2015 | Surgical infections | Observational Study | Factors Affecting Surgical Site Infection Rates in Hepatobiliary Surgery | 2+ |
| 26573175 | Matsumura | 2016 | World J Surg | Observational Study | High Rate of Organ/Space Surgical Site Infection After Hepatectomy with Preexisting Bilioenteric Anastomosis | 2+ |
| 24464624 | Meguro | 2014 | J Hepatobiliary Pancreat Sci | Observational Study | Highest intraoperative lactate level could predict postoperative infectious complications after hepatectomy, reflecting the Pringle maneuver especially in chronic liver disease | 2+ |
| 35618490 | Ruzzenente | 2022 | Surgery | Observational Study | Infectious complications after surgery for perihilar cholangiocarcinoma: A single Western center experience | 2+ |
| 22273719 | Sadamori | 2013 | J Hepatobiliary Pancreat Sci | Observational Study | Risk factors for organ/space surgical site infection after hepatectomy for hepatocellular carcinoma in 359 recent cases | 2+ |
| 28637037 | Shirata | 2018 | Digestive surgery | Observational Study | Surgical Site Infection after Hepatectomy for Hepatocellular Carcinoma | 2+ |
| 30001844 | Takahashi | 2018 | Journal of Infection and Chemotherapy | Observational Study | Risk factors for surgical site infection after major hepatobiliary and pancreatic surgery | 2+ |
| 37086085 | Wang | 2023 | Int Wound J | Observational Study | Surgical site wound infection and pain after laparoscopic repeat hepatectomy for recurrent hepatocellular carcinoma | 2+ |
| 33948991 | Chen | 2021 | Journal of gastroenterology and hepatology | Observational Study | Three-day postoperative antibiotics reduces post-hepatectomy infection rate in hepatitis B virus-related hepatocellular carcinoma | 2+ |

| **Q4: What is the role of antibiotic treatment in cases of postoperative fever of unknown origin and no suspicion of infection**  **or sepsis following liver resection? (n=6)** | | | | | | | |
| --- | --- | --- | --- | --- | --- | --- | --- |
| **PMID** | | **First author** | **Year** | **Journal** | **Type** | **Title** | **LOE** |
| 34623761 | Gupta | 2022 | J Hepatobiliary Pancreat Sci | RCT | Randomized control trial on perioperative antibiotic prophylaxis in live liver donors: Are three doses enough? | 1+ |  |
| 37939273 | Karvellas | 2024 | Hepatology | Practice Guidance | AASLD Practice Guidance on Acute-on-chronic liver failure and the management of critically ill patients with cirrhosis | 1+ |  |
| 30064539 | Bednarsch | 2021 | Scientific Reports | Observational Study | Bacterial bile duct colonization in perihilar cholangiocarcinoma and its clinical significance | 2+ |  |
| [35625294](https://pubmed.ncbi.nlm.nih.gov/35625294/) | Murtha-Lemekhova | 2022 | Antibiotics | Systematic Review and Meta-Analysis | Routine Postoperative Antibiotic Prophylaxis Offers No Benefit after Hepatectomy—A Systematic Review and Meta-Analysis | 1++ |  |
| 28540775 | Tan | 2017 | International Journal of Hyperthermia | Observational Study | Preventing intrahepatic infection after ablation of liver tumours in biliary-enteric anastomosis patients | 2+ |  |
| 28371248 | Okamura. | 2017 | J Hepatobiliary Pancreat Sci | RCT | Randomized controlled trial of perioperative antimicrobial therapy based on the results of preoperative bile cultures in patients undergoing biliary reconstruction | 1++ |  |

Group 3: Prehabilitation, nutrition, mobilisation

Specific additional keywords:

Hepatectomy/ OR (exp Liver/su NOT Liver transplantation/) OR (Hepatectom* OR Hemihepatectom* OR ((Hepatic OR Liver) ADJ3 (surger* OR surgical OR operation* OR resection* OR segmentectom* OR lobectom*))).ab,ti,kf.) AND (Preoperative Exercise/ OR ((exp Exercise/ OR exp Nutritional Support/ OR Nutritional Status/ OR Diet Therapy/ OR exp Dietary Supplements/ OR Immunonutrition Diet/ OR exp Diet/ OR exp Nutritional Support/ OR exp Arginine/ OR exp Glutamine/ OR exp Fatty Acids, Omega-3/ OR Diet, Carbohydrate Loading/ OR exp Carbohydrates/ OR Enteral Nutrition/ OR exp Malnutrition/ OR exp Muscular Atrophy/ OR exp Overweight/ OR exp Cognitive Behavioral Therapy/ OR exp Relaxation Therapy/ OR Smoking Cessation/ OR Smoking Reduction/ OR "Tobacco Use Cessation"/ OR ("physical activit*" OR exercis* OR ((resistance OR endurance OR cardio* OR respiratory) ADJ3 training) OR walking OR climbing OR nutrition* OR diet OR dietary OR Immunonutrition* OR arginine OR glutamine OR "omega 3" OR malnutrition OR malnourished OR (oral ADJ3 supplement*) OR ((drink* OR beverage* OR oral OR loading OR diet) ADJ3 carbohydrate*) OR (feeding ADJ3 tube*) OR "muscle atrophy*" OR myoatrophy OR amyotrophy OR sarcopeni* OR sarcopaeni* OR overweight OR "over weight" OR obesity OR obese OR "psycho educati*" OR psychoeducati* OR "psychological intervention*" OR ("cognitive behavio*" ADJ (therap* OR treatment OR intervention*)) OR (relaxation ADJ3 (training OR techniques OR technic* OR method* OR therap*)) OR (smoking ADJ3 (cessation OR reduction OR decrease OR abstinen*))).ab,ti,kf.) AND ("Preoperative Period"/ OR "Preoperative Care"/ OR (preoperative* OR perioperative OR "peri operative" OR presurgery OR presurgical OR preadmission OR candidate* OR ((before OR prior) ADJ5 (surger* OR surgical OR operation OR operative OR resection OR segmentectom* OR lobectom* OR hepatectomy OR hemihepatectomy)) OR (pre ADJ3 (surger* OR surgical OR operation OR operative OR resection OR segmentectom* OR lobectom* OR Hepatectomy OR hemihepatectomy))).ab,ti,kf.)) OR (exp * Malnutrition/ OR * Nutritional Status/ OR exp * Muscular Atrophy/ OR exp * Overweight/ OR (malnutrition OR malnourished OR "muscle atrophy*" OR myoatrophy OR amyotrophy OR sarcopeni* OR sarcopaeni* OR overweight OR "over weight" OR obesity OR obese).ti,kf.) OR (pre-habilitation OR prehabilitation OR "medical optimization" OR ((pre-operative OR preoperative) ADJ5 (rehabilitation OR habilitation OR conditionning))).ab,ti,kf.) AND English.lg. NOT (exp animals/ NOT humans/) NOT (editorial/ or letter/)

Number of initially retrieved search results: 2183

Excluded studies after screening by working group and steering committee: 2031

Final number of studies: 53

| **Q1: What is the value of a prehabilitation program in patients undergoing hepatectomy?**  **All studies were included in the expert panel commentaries of the manuscript** |
| --- |

| **Q2: What is the influence of preoperative body composition for outcomes in patients undergoing hepatectomy?**  **All studies were included in the expert panel commentaries of the manuscript** |
| --- |

| **Q3: What is the role of fitness screenings and assessments in patients undergoing hepatectomy?**  **All studies were included in the expert panel commentaries of the manuscript** |
| --- |

| **Q4: Does perioperative immunonutrition offer a benefit in patients undergoing hepatectomy?**  **(n=3)** | | | | | | |
| --- | --- | --- | --- | --- | --- | --- |
| **PMID** | **First author** | **Year** | **Journal** | **Type** | **Title** | **LOE** |
| 28385477 | Weimann | 2017 | Clin Nutr | Review | ESPEN guideline: clinical nutrition in surgery. | 1+ |
| 27112357 | Zhao | 2016 | J Investig Med | Systematic Review and Meta-Analysis | Enteral nutrition versus parenteral nutrition after major abdominal surgery in patients with gastrointestinal cancer: a systematic review and meta analysis. | 1+ |
| 25792846 | Gao | 2015 | Onco Targets Ther. | Systematic Review and Meta-Analysis | Early enteral and parenteral nutritional support after hepatectomy in patients with hepatic carcinoma: a systematic review and meta-analysis. | 1+ |

| **Q5: What is the role of prokinetic policies to improve time to first bowel movement/full standard diet?**  **All studies were included in the expert panel commentaries of the manuscript** |
| --- |

| **Q6: When and how should artificial nutrition be added to oral nutrition in the postoperative period?**  **All studies were included in the expert panel commentaries of the manuscript** |
| --- |

| **Q7: How should (early) postoperative mobilization after hepatectomy be defined?**  **All studies were included in the expert panel commentaries of the manuscript** |
| --- |

Group 4: Post-hepatectomy bile leak including bilioenteric anastomosis leaks

Specific additional keywords:

TS=((liver OR hepat*) NEAR/3 (surgery OR surgeries OR resection* OR lobectom* OR metastasectom*)) OR TS=(hepatectom* OR hemihepatectom* OR trisectionectom*)

TS=((post-hepatectom* OR postoperat* OR post-operat* OR postsurg* OR post-surg* OR “after hepatectomy” OR “after partial hepatectomy” OR “after operation” OR “after surgery” OR “following hepatectomy” OR “following partial hepatectomy” OR “following operation” OR “following surgery”) NEAR/9 (bile or biliar* OR bilio*) NEAR/3 (leak* or anastomo* OR fistula*)) OR TS=((leak* or anastomo*) NEAR/6 intraoperative NEAR/3 ("white test" OR "indocyanine green" OR "methylene blue" OR ICG))

Number of initially retrieved search results: 951

Excluded studies after screening by working group and steering committee: 904

Final number of studies: 47

| **Q1 - Is there a role for intraoperative bile leak test (i.e. white test, methylene blue, ICG…) after hepatectomy without bilio-enteric anastomosis? (n=8)** | | | | | | |  |
| --- | --- | --- | --- | --- | --- | --- | --- |
| **PMID** | **First author** | **Year** | **Journal** | **Type** | **Title** | **LOE** | |
| 32807583 | Tuysuz | 2021 | Asian J Surg | Retrospective study | The role of Intraoperative cholangiography (IOC) and methylene blue tests in reducing bile leakage after living donor hepatectomy | 2- | |
| 34600501 | Chopinet | 2021 | BMC Surg | Case series | The white test for intraoperative screening of bile leakage: a potential trigger factor for acute pancreatitis after liver resection-a case series | 4 | |
| 31182455 | Tanaka | 2019 | BMJ Open | Prospective single-arm clinical trial | Efficacy of the bile leak test using contrast-enhanced intraoperative ultrasonic cholangiography in liver resection: a study protocol for a non-randomised, prospective, off-label, single-arm trial | 1+ | |
| 24246619 | Zimmitti | 2013 | J Am Coll Surg | Clinical trial with retrospective matched cohort | Systematic use of an intraoperative air leak test at the time of major liver resection reduces the rate of postoperative biliary complications | 2+ | |
| 22547901 | Leelawat | 2012 | HPB Surg | Prospective controlled trial | Evaluation of the white test for the intraoperative detection of bile leakage | 2+ | |
| 21514613 | Kaibori | 2011 | Surgery | Randomised clinical trial | Intraoperative indocyanine green fluorescent imaging for prevention of bile leakage after hepatic resection | 1- | |
| 21552666 | Kayaalp | 2011 | Clinics | Retrospective study | Leakage tests reduce the frequency of biliary fistulas following hydatid liver cyst surgery | 2- | |
| 20637329 | Sakaguchi | 2010 | Am J Surg | Retrospective study | Bile leak test by indocyanine green fluorescence images after hepatectomy | 2+ | |
| 32807583 | Tuysuz | 2021 | Asian J Surg | Retrospective study | The role of Intraoperative cholangiography (IOC) and methylene blue tests in reducing bile leakage after living donor hepatectomy | 2- | |
| 34600501 | Chopinet | 2021 | BMC Surg | Case series | The white test for intraoperative screening of bile leakage: a potential trigger factor for acute pancreatitis after liver resection-a case series | 4 | |
| 31182455 | Tanaka | 2019 | BMJ Open | Prospective single-arm clinical trial | Efficacy of the bile leak test using contrast-enhanced intraoperative ultrasonic cholangiography in liver resection: a study protocol for a non-randomised, prospective, off-label, single-arm trial | 1+ | |
| 24246619 | Zimmitti | 2013 | J Am Coll Surg | Clinical trial with retrospective matched cohort | Systematic use of an intraoperative air leak test at the time of major liver resection reduces the rate of postoperative biliary complications | 2+ | |
| 22547901 | Leelawat | 2012 | HPB Surg | Prospective controlled trial | Evaluation of the white test for the intraoperative detection of bile leakage | 2+ | |
| 21514613 | Kaibori | 2011 | Surgery | RCT | Intraoperative indocyanine green fluorescent imaging for prevention of bile leakage after hepatic resection | 1- | |
| 21552666 | Kayaalp | 2011 | Clinics | Retrospective study | Leakage tests reduce the frequency of biliary fistulas following hydatid liver cyst surgery | 2- | |
| 20637329 | Sakaguchi | 2010 | Am J Surg | Retrospective study | Bile leak test by indocyanine green fluorescence images after hepatectomy | 2+ | |

| **Q2 - Is there a role for drainage after hepatectomy without bilio-enteric reconstruction?**  **All studies were included in the expert panel commentaries of the manuscript** |
| --- |

| **Q3 - What is the value of biliary trans-anastomotic stents in complex bilio-enteric anastomoses in hepatectomy? (n=2)** | | | | | | |
| --- | --- | --- | --- | --- | --- | --- |
| **PMID** | **First author** | **Year** | **Journal** | **Type** | **Title** | **LOE** |
| 30091039 | Collard | 2018 | J Gastrointest Surg | Retrospective study | Duct-To-Duct Biliary Anastomosis with Removable Internal Biliary Stent During Major Hepatectomy Extended to the Biliary Confluence. | 2- |
| 22081253 | Hirano | 2012 | J Hepatobiliary Pancreat Sci | Case series | Techniques of biliary reconstruction following bile duct resection (with video) | 4 |

| **Q4 - Is there a role for routine imaging (computed tomography (CT), magnetic resonance (MR) cholangiography) in high-risk patients for bile leaks?**  **All studies were included in the expert panel commentaries of the manuscript** |
| --- |

| **Q5 - What is the role of endoscopy and interventional radiology in patients with clinically relevant bile leakage after hepatectomy?**  **All studies were included in the expert panel commentaries of the manuscript** |
| --- |

Group 5: Post-hepatectomy haemorrhage (PHH)

Specific additional keywords:

TS=((liver OR hepat*) NEAR/3 (surgery OR surgeries OR resection* OR lobectom* OR metastasectom*)) OR TS=(hepatectom* OR hemihepatectom* OR trisectionectom*) TS=((post-hepatectom* OR postoperat* OR post-operat* OR postsurg* OR post-surg* OR “after hepatectomy” OR “after partial hepatectomy” OR “after operation” OR “after surgery” OR “following hepatectomy” OR “following partial hepatectomy” OR “following operation” OR “following surgery”) NEAR/9 (bile or biliar* OR bilio*) NEAR/3 (leak* or anastomo* OR fistula*)) OR TS=((leak* or anastomo*) NEAR/6 intraoperative NEAR/3 ("white test" OR "indocyanine green" OR "methylene blue" OR ICG))

Number of initially retrieved search results: 860

Excluded studies after screening by working group and steering committee: 800

Final number of studies: 60

| **Q1: What is the role of pre-operative strategies to reduce blood loss associated with liver resection**  **(fat-reducing diet, nutritional changes, hypovolaemic phlebotomy)? (n=2)** | | | | | | |
| --- | --- | --- | --- | --- | --- | --- |
| **PMID** | **First author** | **Year** | **Journal** | **Type** | **Title** | **LOE** |
| 39667380 | Martel | 2024 | Lancet Gast Onc | RCT | Hypovolaemic phlebotomy in patients undergoing hepatic resection at higher risk of blood loss (PRICE-2): a randomised controlled trial | 1+ |

| **Q2: What is the role of anaesthetic management to reduce blood loss in patients undergoing hepatectomy? (n=1)** | | | | | | |
| --- | --- | --- | --- | --- | --- | --- |
| **PMID** | **First author** | **Year** | **Journal** | **Type** | **Title** | **LOE** |
| 39158894 | Karanicolas | 2024 | JAMA | RCT | Tranexamic Acid in Patients Undergoing Liver Resection: The HeLiX Randomized Clinical Trial | 1+ |

| **Q3: What is the value of haemostatic agents to reduce blood loss in patients undergoing hepatectomy?**  **All studies were included in the expert panel commentaries of the manuscript** |
| --- |

| **Q4: Does Minimally-invasive surgery influence blood loss in patients undergoing hepatectomy? (n=1)** | | | | | | |
| --- | --- | --- | --- | --- | --- | --- |
| **PMID** | **First author** | **Year** | **Journal** | **Type** | **Title** | **LOE** |
| 38640453 | Fichtinger | 2024 | J Clin Onc | RCT | Laparoscopic Versus Open Hemihepatectomy: The ORANGE II PLUS Multicenter Randomized Controlled Trial | 1+ |

| **Q5: Is there an influence of parenchymal transection technique on blood loss?**  **All studies were included in the expert panel commentaries of the manuscript** |
| --- |

| **Q6: What is the role for intra-operative liver inflow occlusion in reducing intra-operative blood loss?**  **All studies were included in the expert panel commentaries of the manuscript** |
| --- |

Group 6: Post-hepatectomy liver failure (PHLF)

Specific additional keywords:

"TS=((liver OR hepat*) NEAR/3 (surgery OR surgeries OR resection* OR lobectom* OR metastasectom*)) OR TS=(hepatectom* OR hemihepatectom* OR trisectionectom*) "TS=((liver NEAR/3 (failure OR dysfunction OR insufficiency) NEAR/6 (post-hepatectom* OR post-operat* OR post-surg* OR ""after hepatectomy"" OR ""after partial hepatectomy"" OR ""after operation"" OR ""after surgery"" OR ""following hepatectomy"" OR ""following partial hepatectomy"" OR ""following operation"" OR ""following surgery"")) OR PHLF) OR TS=(hepatic NEAR/3 (failure OR dysfunction OR insufficiency) NEAR/6 (post-hepatectom* OR post-operat* OR post-surg* OR ""after hepatectomy"" OR ""after partial hepatectomy"" OR ""after operation"" OR ""after surgery"" OR ""following hepatectomy"" OR ""following partial hepatectomy"" OR ""following operation"" OR ""following surgery"")) OR TI=(mortality AND hepatectom*)

"

Number of initially retrieved search results: 961

Excluded studies after screening by working group and steering committee: 710

Final number of studies: 75

| **Q1: Is there a clinical relevance to sub classify PHLF by the main underlying pathomechanism?**  **Q2: Should patients suffering from PHLF be monitored beyond 90 days after hepatectomy? (n= 16)** | | | | | | |
| --- | --- | --- | --- | --- | --- | --- |
| **PMID** | **First author** | **Year** | **Journal** | **Type** | **Title** | **LOE** |
| 38225867 | Vitello | 2024 | Journal of surgical oncology | Retrospective study | Establishing the clinical relevance of grade A post-hepatectomy liver failure | 2+ |
| 29373299 | Yu | 2018 | HBP (Oxford) | Retrospective study | Post-operative delayed elevation of ALT correlates with early death in patients with HBV-related hepatocellular carcinoma and Post-hepatectomy Liver Failure | 2+ |
| 29179518 | Zheng | 2017 | Oncotarget | Retrospective study | Reassessment of different criteria for diagnosing post-hepatectomy liver failure: a single-center study of 1683 hepatectomy | 2+ |
| 25243550 | Skrzypczyk | 2014 | Annals of surgery | Retrospective study | Relevance of the ISGLS definition of posthepatectomy liver failure in early prediction of poor outcome after liver resection: study on 680 hepatectomies | 2+ |
| 27506991 | van Mierlo | 2016 | HBP (Oxford) | Retrospective study | Validation of the peak bilirubin criterion for outcome after partial hepatectomy | 2- |
| 29421802 | Kawamura | 2019 | Digestive surgery | Retrospective study | Postoperative Liver Failure Criteria for Predicting Mortality after Major Hepatectomy with Extrahepatic Bile Duct Resection. | 2+ |
| 23423450 | Kim | 2013 | World J Surg | Retrospective study | Early predictor of mortality due to irreversible posthepatectomy liver failure in patients with hepatocellular carcinoma | 2+ |
| 26063080 | Lafaro | 2015 | Journal of gastrointestinal surgery | Review | Defining Post Hepatectomy Liver Insufficiency: Where do We stand? | 1- |
| 30196769 | Birgin | 2019 | Scandinavian journal of surgery | Retrospective study | Evaluation of the New ISGLS Definitions of Typical Posthepatectomy Complications." | 2- |
| 34913896 | Calthorpe | 2023 | Annals of surgery | Retrospective study | Using the Comprehensive Complication Index to Rethink the ISGLS Criteria for Post-hepatectomy Liver Failure in an International Cohort of Major Hepatectomies. | 2+ |
| 33708932 | Li | 2021 | Annals of translational medicine | Retrospective study | A cohort study of hepatectomy-related complications and prediction model for postoperative liver failure after major liver resection in 1,441 patients without obstructive jaundice | 3 |
| 31792904 | Li | 2020 | Journal of gastrointestinal surgery | Retrospective study | Is It Feasible to Standardize a Composite Postoperative Complication Reporting System for Liver Resection | 3 |
| 21689231 | Mayo | 2011 | HBP (Oxford) | Retrospective study | Refining the definition of perioperative mortality following hepatectomy using death within 90 days as the standard criterion | 3 |
| 22722672 | Merad | 2012 | World J Surg | Retrospective study | Prospective evaluation of in-hospital mortality with the P-POSSUM scoring system in patients undergoing major digestive surgery | 3 |
| 29916105 | Merath | 2018 | Journal of gastrointestinal surgery | Retrospective study | Synergistic Effects of Perioperative Complications on 30-Day Mortality Following Hepatopancreatic Surgery | 3 |
| 34334638 | Mueller | 2021 | Annals of surgery | Retrospective study | Perihilar Cholangiocarcinoma - Novel Benchmark Values for Surgical and Oncological Outcomes From 24 Expert Centers | 3 |

| **Q3: Which postoperative markers should be considered to monitor postoperative liver function recovery in patients at high risk of PHLF?**  **Q4: Is there a value for early postoperative markers to predict PHLF? ( n= 35)** | | | | | | |
| --- | --- | --- | --- | --- | --- | --- |
| **PMID** | **First author** | **Year** | **Journal** | **Type** | **Title** | **LOE** |
| 31197694 | Wang | 2020 | Journal of gastrointestinal surgery | Retrospective study | Predictive Value of Intraoperative Indocyanine Green Clearance Measurement on Postoperative Liver Function After Anatomic Major Liver Resection | 2- |
| 38007060 | Wei | 2024 | Journal of thrombosis and haemostasis | Retrospective study | Coagulation factor XIII is a critical driver of liver regeneration after partial hepatectomy | 2- |
| 24298201 | Wiggans | 2013 | HBP (Oxford) | Retrospective study | Renal dysfunction is an independent risk factor for mortality after liver resection and the main determinant of outcome in posthepatectomy liver failure | 2+ |
| 25001260 | Yoshida | 2014 | Annals of nuclear medicine | Retrospective study | Assessment of hepatic functional regeneration after hepatectomy using (99m)Tc-GSA SPECT/CT fused imaging | 2- |
| 23458705 | Roberts | 2013 | HBP (Oxford) | Retrospective study | Kinetics of liver function tests after a hepatectomy for colorectal liver metastases predict post-operative liver failure as defined by the International Study Group for Liver Surgery | 2+ |
| 33369454 | Sawangkajohn | 2020 | APJCP | Retrospective study | Re-Rising of Total Bilirubin Level after Postoperative Day 3 (The V Pattern) Predicting Liver Failure and Survival of Patients who Underwent Hepatectomy for Cholangiocarcinoma | 2+ |
| 35144899 | Silva | 2022 | The surgeon : journal of the Royal Colleges of Surgeons of Edinburgh and Ireland | Retrospective study | Early derangement of INR predicts liver failure after liver resection for hepatocellular carcinoma | 2+ |
| 24830898 | Squires | 2014 | HBP (Oxford) | Retrospective study | Hypophosphataemia after major hepatectomy and the risk of post-operative hepatic insufficiency and mortality: an analysis of 719 patients | 2- |
| 31669198 | Truant | 2020 | HBP (Oxford) | Prospective study | Asymmetric kinetics of volume and function of the remnant liver after major hepatectomy as a key for postoperative outcome - A case-matched study | 2+ |
| 25581073 | Thomas | 2015 | HBP (Oxford) | Prospective study | Intraoperative simulation of remnant liver function during anatomic liver resection with indocyanine green clearance (LiMON) measurements | 1- |
| 36428685 | Felli | 2022 | Cancers | Prospective study | Hyperspectral Imaging in Major Hepatectomies: Preliminary Results from the Ex-Machyna Trial." | 2+ |
| 26516057 | Golriz | 2016 | Clinics and research in hepatology and gastroenterology | Review | Small for Size and Flow (SFSF) syndrome: An alternative description for posthepatectomy liver failure." | 1- |
| 25611592 | Haegele | 2015 | PloS one | Retrospective study | Deficiency in thrombopoietin induction after liver surgery is associated with postoperative liver dysfunction | 2- |
| 26938452 | Helling | 2016 | PloS one | Retrospective study | Plasma Glutamine Concentrations in Liver Failure | 2- |
| 26385577 | Herbert | 2015 | HBP (Oxford) | Retrospective study | Early trends in serum phosphate and creatinine levels are associated with mortality following major hepatectomy." | 2+ |
| 31549011 | Imai | 2019 | Annals of gastroenterological surgery | Retrospective study | Elevation of Mac-2 binding protein glycosylation isomer after hepatectomy is associated with post-hepatectomy liver failure, total Pringle time, and renal dysfunction | 2+ |
| 26058324 | Jara | 2015 | HBP (Oxford) | Retrospective study | Reductions in post-hepatectomy liver failure and related mortality after implementation of the LiMAx algorithm in preoperative work-up: a single-centre | 2+ |
| 38150185 | Baumgartner | 2024 | BJS | Retrospective study | Comparing the accuracy of prediction models to detect clinically relevant post-hepatectomy liver failure early after major hepatectomy | 2+ |
| 38305104 | Duque | 2024 | Blood coagulation & fibrinolysis | Prospective study | The link between high factor VIII to protein C ratio values and poor liver function after major hepatectomy | 2+ |
| 24836954 | Etra | 2014 | HBP (Oxford) | Retrospective study | Early identification of patients at increased risk for hepatic insufficiency, complications and mortality after major hepatectomy." | 1- |
| 32065510 | Connolly | 2020 | Journal of hepato-biliary-pancreatic sciences | Systematic Review | Systematic review on peri-operative lactate measurements to predict outcomes in patients undergoing liver resection." | 1- |
| 38099865 | Brunnthaler | 2024 | Hepatology communications | Prospective study | Intrahepatic neutrophil accumulation and extracellular trap formation are associated with posthepatectomy liver failure | 1- |
| 34601569 | Lee | 2021 | BJS open | Observational Study | Acute kidney injury following hepatectomy and its impact on long-term survival for patients with hepatocellular carcinoma | 3 |
| 28182632 | Li | 2017 | PloS one | Systematic Review | Clinical outcomes of patients with and without diabetes mellitus after hepatectomy: A systematic review and meta-analysis | 2 |
| 37428411 | Liao | 2023 | Updates in surgery | Retrospective study | Sequential transcatheter arterial chemoembolization and portal vein embolization before hepatectomy for the management of patients with hepatocellular carcinoma: a systematic review and meta-analysis. | 3 |
| 37329363 | Mangieri | 2023 | Langenbeck's archives of surgery | Retrospective study | Risk factors and outcomes for cholangitis after hepatic resection. | 3 |
| 27121234 | Margonis | 2016 | Journal of gastrointestinal surgery | Retrospective study | Impact of Perioperative Phosphorus and Glucose Levels on Liver Regeneration and Long-term Outcomes after Major Liver Resection | 3 |
| 26736156 | Newhook | 2015 | The American surgeon | Retrospective study | Impact of Postoperative Venous Thromboembolism on Postoperative Morbidity, Mortality, and Resource Utilization after Hepatectomy | 3 |
| 34694377 | Niederwieser | 2021 | BJS | Retrospective study | Early postoperative arterial lactate concentrations to stratify risk of post-hepatectomy liver failure | 3 |
| 20583434 | Okano | 2010 | Hepato-gastroenterology | Retrospective study | ADAMTS13 activity decreases after hepatectomy, reflecting a postoperative liver dysfunction | 4 |
| 36157153 | Parwaiz | 2022 | Journal of clinical and experimental hepatology | Retrospective study | Does ALT Correlate with Survival After Liver Resection for Colorectal Liver Metastases? | 3 |
| 27275463 | Hallet | 2016 | Hepatobiliary surgery and nutrition | Retrospective study | Hypophosphatemia and recovery of post-hepatectomy liver insufficiency. | 2- |

| **Q5: Which treatments are available to support hepatic regeneration in PHLF? ( n= 18)** | | | | | | |
| --- | --- | --- | --- | --- | --- | --- |
| **PMID** | **First author** | **Year** | **Journal** | **Type** | **Title** | **LOE** |
| 37059100 | Wang | 2023 | Cell stem cell | Retrospective study | Reversal of liver failure using a bioartificial liver device implanted with clinical-grade human-induced hepatocytes | 2- |
| 23811794 | Yang | 2013 | World J Surg | Retrospective study | Risk factors of hospital mortality after re-laparotomy for post-hepatectomy hemorrhage | 2+ |
| 23458723 | Robinson | 2013 | HBP (Oxford) | Retrospective study | N-acetylcysteine administration does not improve patient outcome after liver resection | 2- |
| 34053931 | Ryu | 2021 | Annals of hepato-biliary-pancreatic surgery | Retrospective study | Therapeutic plasma exchange as an effective salvage measure for post-hepatectomy hepatic failure: A case report | 2- |
| 35067465 | Sparrelid | 2022 | HBP (Oxford) | Retrospective study | Liver transplantation in patients with post-hepatectomy liver failure - A Northern European multicenter cohort study | 2+ |
| 30929063 | Thorsen | 2019 | Langenbeck's archives of surgery | Retrospective study | Liver transplantation as a lifesaving procedure for posthepatectomy liver failure and iatrogenic liver injuries | 2- |
| 28028637 | Fuji | 2017 | Surgery today | Phase I clinical trial | Phase I clinical trial of olprinone in liver surgery | 2+ |
| 3337862 | Gavriilidis | 2020 | Chirurgia | Retrospective study | Effectiveness of Terlipressin on Modulation of Portal Vein Pressure after Hepatic Resections in Non-Cirrhotic Patients. | 2- |
| 36000747 | Gilg | 2022 | Scand J Surg | Pilot study | Molecular adsorbent recirculating system treatment in patients with post-hepatectomy liver failure: Long-term results of a pilot study | 2+ |
| 29619422 | Gilg | 2018 | Hepatol Commun. | Phase I clinical trial | The molecular adsorbent recirculating system in posthepatectomy liver failure: Results from a prospective phase I study." | 2+ |
| 27302646 | Grendar | 2016 | Journal of surgical oncology | RCT | Effect of N-acetylcysteine on liver recovery after resection: A randomized clinical trial | 1+ |
| 37845430 | Iida | 2023 | Langenbeck's archives of surgery | Prospective study | Effect of early administration of tolvaptan on pleural effusion post-hepatectomy. | 1- |
| 31680011 | Kohler | 2020 | HBP (Oxford) | RCT | Effectiveness of terlipressin for prevention of complications after major liver resection - A randomized placebo-controlled trial | 1- |
| 37749790 | Azoulay | 2024 | Transplantation | Systematic Review | Rescue Liver Transplantation for Posthepatectomy Liver Failure: A Systematic Review and Survey of an International Experience." | 1- |
| 31388643 | Dasari | 2019 | BJS open | Propensity-matched analysis | Propensity-matched analysis of the influence of perioperative statin therapy on outcomes after liver resection | 2+ |
| 31626819 | Linecker | 2020 | J hep | RCT | Perioperative omega-3 fatty acids fail to confer protection in liver surgery: Results of a multicentric, double-blind, randomized controlled trial." | 2 |
| 33471344 | Moghadamyeghaneh | 2021 | Updates in surgery | Retrospective study | Outcome of bile leakage following liver resection with hepaticojejunostomy for liver cancer | 4 |

**Supplementary Figures**

Quality Control by Validation Committee
